# Visibility matters during wayfinding in the vertical

**DOI:** 10.1038/s41598-021-98439-1

**Published:** 2021-09-23

**Authors:** Michal Gath-Morad, Tyler Thrash, Julia Schicker, Christoph Hölscher, Dirk Helbing, Leonel Enrique Aguilar Melgar

**Affiliations:** 1 Chair of Cognitive Science, ETH Zurich, Zurich, Switzerland; 2 Computational Social Science, ETH Zurich, Zurich, Switzerland; 3 Data Science, Systems and Services Laboratory, ETH Zurich, Zurich, Switzerland; 4 Department of Biology, Miami University, Oxford, USA

**Keywords:** Psychology, Human behaviour, Civil engineering

## Abstract

Visibility is the degree to which different parts of the environment can be observed from a given vantage point. In the absence of previous familiarity or signage, the visibility of key elements in a multilevel environment (e.g., the entrance, exit, or the destination itself) becomes a primary input to make wayfinding decisions and avoid getting lost. Previous research has focused on memory-based wayfinding and mental representation of 3D space, but few studies have investigated the direct effects of visibility on wayfinding. Moreover, to our knowledge, there are no studies that have explicitly observed the interaction between visibility and wayfinding under uncertainty in a multilevel environment. To bridge this gap, we studied how the visibility of destinations, as well as the continuity of sight-lines along the vertical dimension, affects unaided and goal-directed wayfinding behavior in a multilevel desktop Virtual Reality (VR) study. We obtained results from a total of 69 participants. Each participant performed a total of 24 wayfinding trials in a multilevel environment. Results showcase a significant and nonlinear correlation between the visibility of destinations and wayfinding behavioral characteristics. Specifically, once the destination was in sight, regardless of whether it was highly or barely visible, participants made an instantaneous decision to switch floors and move up towards the destination. In contrast, if the destination was out-of-sight, participants performed ‘visual exploration’, indicated by an increase in vertical head movements and greater time taken to switch floors. To demonstrate the direct applicability of this fundamental wayfinding behavioral pattern, we formalize these results by modeling a visibility-based cognitive agent. Our results show that by modeling the transition between exploration and exploitation as a function of visibility, cognitive agents were able to replicate human wayfinding patterns observed in the desktop VR study. This simple demonstration shows the potential of extending our main findings concerning the nonlinear relationship between visibility and wayfinding to inform the modeling of human cognition.

## Introduction

The majority of wayfinding studies conducted in the last 70 years^1–3^ have focused on processes of spatial knowledge acquisition and subsequent neural encoding of place, grid, and head direction cells in the brain^4,5^. According to the cognitive map hypothesis^6^, these cells form the substrate underlying an internal representation of previously navigated environments that supports memory-based wayfinding and other intelligent spatial behaviors such as short-cutting. This type of internal representation is assumed to have a primary role during unaided wayfinding (i.e., without the use of external symbolic information from maps, signage, or Information and Communication Technologies)^7^ towards familiar destinations^5^, and plays a limited role during unaided wayfinding towards unfamiliar destinations^8,9^. For unfamiliar destinations, individuals do not have any previous information about the navigated environment beyond the perception of their immediate surroundings and are thus unable to use an internal representation to support wayfinding. However, these individuals may still have to make wayfinding decisions based on this limited information in order to find their way. In such situations, visibility (i.e., the degree to which different parts of the environment can be observed from a given vantage point) becomes a primary consideration for the navigator^8–12^. Visibility may also help the navigator avoid the negative consequences of getting lost, including stress, frustration, and delays.

Wayfinding in multilevel environments pose particular challenges with respect to visibility, especially in complex buildings with numerous entrances, destinations, exits, and barriers^12–14^. For example, the positioning of a building’s entrance with respect to key destinations often disregards visibility towards these destinations, despite providing critical information for the support of wayfinding by novice occupants. Moreover, barriers such as floor slabs, walls, elevators, stairs, and shafts may block visibility towards key building elements along both horizontal and vertical dimensions. Empirical research on wayfinding in multilevel buildings highlights that people tend to find their way less efficiently and commit more errors in the case of ‘between-floor tasks’ (i.e., tasks in which the start location and destination are on different floors) compared to ‘within-floor tasks’ (i.e., tasks in which the start location and destination are on the same floor)^15–22^. These difficulties are typically attributed to the overall fit between the participants’ search strategies and an environment’s configuration^12,18^, but much of this literature has yet to explicitly test for the potential effects of visibility on wayfinding through a multilevel, unfamiliar building^11,12,19^.

The importance of the vertical dimension for wayfinding has also been investigated in terms of anisotropies^5,20,21^ and hierarchical environments^12,22–26^ in 3D space. Both of these research areas emphasize the roles of different aspects of spatial representation for learning during wayfinding. Specifically, research on anisotropies has demonstrated evidence of higher neuronal sensitivity for locations and movements along the horizontal dimension in comparison to the vertical dimension^15^. These findings led to Jeffery et al. bio-coded cognitive map hypothesis, postulating that representations of 3D space are subject to anisotropy, such that information along the horizontal dimension is encoded more precisely than information in the vertical space^5^. Research conducted in multilevel buildings present supporting evidence of this hypothesis^12,17–21^. Although this extensive body of research provides important insights into mental representations of 3D spaces, far fewer studies have investigated how visibility of 3D space directly affects wayfinding behavior and performance.

In most of the previous literature on visibility and wayfinding, the visibility afforded by an environmental layout is analyzed with respect to its geometrical or topological properties and then correlated with aggregated wayfinding behavior. Indeed, the Space Syntax community has contributed many examples of the development and validation of topological measures that capture the degree of inter-visibility between various locations in the environment (i.e., whether one location is visible from another location and vice versa)^27^. These topological graph-based measures are often correlated with movement patterns to assess an environment’s capacity to accommodate a particular intended function^11,28^. In addition, geometrical properties of a visible space may be captured by the concept of isovists. An two-dimensional isovist is a horizontal slice (i.e., polygon) through an environment that represents the three-dimensional view volume from a human’s perspective at a specific point in time and space^29^. This single polygon can then be used to derive quantitative geometrical properties of visible space, including area, perimeter, and various other derivative measures. Several studies have used isovists to describe the relationship between visibility and wayfinding^19,30^, but most of these efforts emphasized the connection between wayfinding behavior and measures of overall visibility (e.g., the size of the visible area or volume within the environment) or between wayfinding behavior and specific geometrical and topological features of visible space. This approach disregards information on ‘what’ is visible and the relevance of this visible information to a particular wayfinding task (e.g., visibility towards the destination).

Recently, He et al.^31^ explicitly connected visibility and wayfinding in a desktop VR setting. They hypothesized that the reduced visibility caused by barriers can play a major role in the accumulation of error during spatial updating and the encoding of spatial relations. Participants learned an environment by wayfinding with either ‘X-ray’ vision (i.e., barriers became translucent to maximize visibility) or regular vision (i.e., barriers occluded visibility) and were then tested on their spatial knowledge of that environment. Their results revealed that participants with a high level of self-reported ‘sense of direction’ who learned the environment with ‘X-ray’ vision had better performance in wayfinding and pointing tasks than participants who learned an environment in which visibility was often occluded by barriers. Although this study provides a useful example of how barriers (i.e., occlusions to visibility) may affect wayfinding, the study was conducted in a single-level environment with highly contrasted visibility conditions. Similar to previous research on hierarchical environments^12,22–26^, He et al.^31^ also investigated the effect of visibility on spatial representation and learning during wayfinding rather than the effect of visibility on spatial decision-making in an unfamiliar environment.

**Figure 1:**
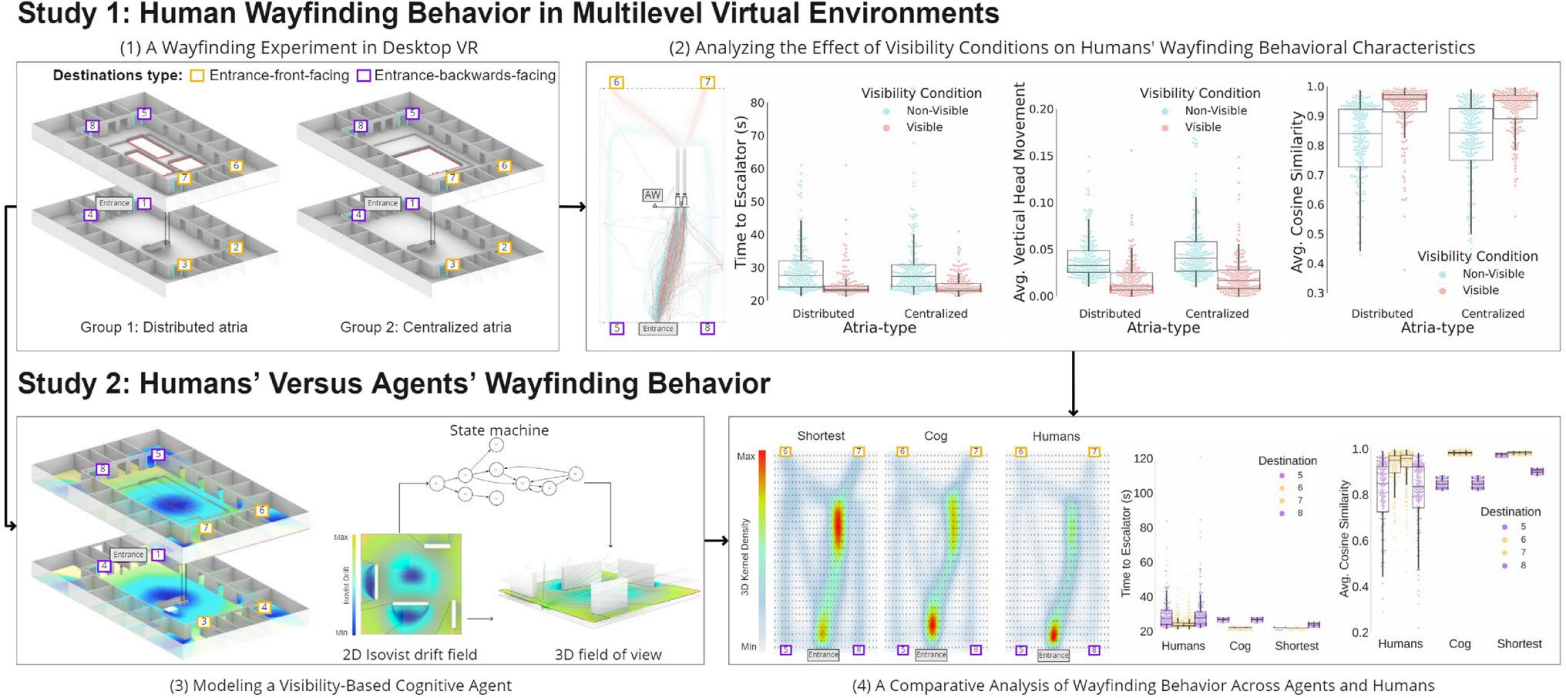
An overview of the research design and the links between the different research stages; Study 1 includes two stages: (1) A wayfinding experiment in desktop VR under two systematically varied multilevel environments and (2) An analysis of the experiments’ results to quantify the effects of visibility on wayfinding behavioral characteristics. On the basis of this analysis, we conducted study 2 that included two additional research stages: (3) Modeling of a visibility-based cognitive agent that aims to capture observed human wayfinding behavior and finally stage (4) A comparative analysis that analyzed the similarity between agents’ and humans’ wayfinding behavior in respective environments (i.e., distributed versus centralized atria-type). Software used to create this figure: Python^34^ (version 3.5.8), https://www.python.org/downloads/release/python-358/; Seaborn^35^ (version 0.11.1), https://seaborn.pydata.org/index.html;Matplotlib^36^ (version 3.3.2), https://matplotlib.org/3.3.2/users/installing.html; Rhino 6 for Windows (Version 6)^37^, https://www.rhino3d.com/download/; QGis^38^ (version 3.16), https://qgis.org/en/site/forusers/download.html.

Further research is needed for researchers to gain a nuanced understanding of how changes in the visibility of particular elements (e.g., destinations) during wayfinding affects the search for those elements. Such variations in visibility are typical for real-world wayfinding tasks such as finding a particular room in a multilevel building. The present study aims to fill these gaps to better understand the relationship between visibility and wayfinding in complex environments. We present results from two interrelated studies (for an overview, see Fig. 1). The first study was conducted in desktop VR and included 69 participants. In contrast to ‘Immersive VR’ that typically employs a Head Mounted Display (HMD) in which the user is perceptually surrounded by a virtual environment^32^, ‘Desktop VR’ displays the virtual environment on a computer monitor, and interaction with the virtual environment is often provided by handheld interfaces such as a mouse and keyboard^33^. The aim of the study was to investigate how the visibility of a destination and the continuity of visibility (e.g., intermittently occluded by barriers) in the vertical dimension affects performance and spatial behavior during goal-directed wayfinding in an unfamiliar multilevel environment.

Our first hypothesis was that a higher degree of visibility towards upper floor destinations (compared to a lower degree of destination visibility) would significantly improve wayfinding performance and affect other behavioral characteristics such as head movements. Consistent with previous research, our second hypothesis was that wayfinding performance would improve when visibility in the vertical dimension was continuous compared to when visibility was fragmented by barriers. To test these hypotheses, we designed two environments in which we systematically varied two aspects of visibility: (1) the locations of destinations in the multilevel environment and (2) the continuity of visibility in the vertical dimension as a consequence of environmental geometry. Participants’ initial positions and initial heading were fixed across trials, and they were requested to find their way towards destinations that were either ‘entrance-forward-facing’ (naturally more visible from the initial position) or ‘entrance-backwards-facing’ (naturally less visible from the initial position). In addition, the atria design varied across two otherwise identical multilevel buildings. One of these virtual buildings had a single atrium supporting relativity continuous visibility in the vertical dimension, and the other virtual building had three distributed atria that caused visibility to be relativity fragmented by the floor connecting the different atria voids. Participants were randomly assigned to one of the two buildings (i.e., distributed or centralized atria) and had to complete a total of 24 wayfinding trials. Our results confirmed our first hypothesis. Specifically, the experiment revealed an unexpected drastic wayfinding behavioral change that depended on the visibility of the destination, demonstrating a nonlinear relationship between the visibility of the destination and wayfinding behavioral characteristics.

To formally demonstrate how these findings may be used to model wayfinding behavior, we also present a second study on the development of a visibility-based cognitive agent. The results from simulation experiments, based on the same virtual environment, showcase significant similarity between visibility-based cognitive agents and human participants. As a control, we also observed stark contrasts between human participants and classical shortest-path agents (i.e., that do not account for visibility). These results highlight the importance of modeling the dynamics between visibility and wayfinding to constrain predictions of spatial behavior under uncertainty in complex and multilevel environments.

## Results

### Study 1: Human wayfinding behavior in multilevel virtual environments

We obtained results from 69 participants. Each participant performed 8 wayfinding trials, repeated in a randomized order over three blocks totalling 24 wayfinding trials (i.e., the same eight destinations were used across blocks at a random order). The starting location for each trial is referred to as the entrance, positioned at the same location on the first floor for both the distributed and centralized building. Each of the eight destinations was located on either the first or second floor, left or right of the entrance, and was either ‘entrance-forward-facing’ or ‘entrance-backwards-facing’ (see Fig. 1). The locations of the destinations were identical in both virtual buildings, such that atria-type was the only difference between groups. Since we were only interested in wayfinding ‘between floors’, the first-floor data was not analyzed. Nonetheless, the inclusion of first-floor destinations was necessary, see the “Methods” section for more detail. This exclusion resulted in the analysis of 12 trials for each of the 69 participants (i.e., 828 trials in total).

Three wayfinding behavioral measures were calculated from this data, considering a specific analysis window from the beginning of each trial until the bottom step of the escalator: (1)‘Time to Escalator’ (2) ‘Average Vertical Head Movement’ and (3) ‘Average Cosine Similarity’. We used this specific analysis window instead of the entire path from entrance to destination because of differences in the distance of each second-floor destination from the escalator. In contrast, the bottom of the escalator was always the same distance from the entrance and represented the location at which participants must have decided to switch floors. Additionally, We were also focused on the effect of visibility via the different atria types from the first floor to the second floor.

‘Time to Escalator’ was defined as the time required to reach the bottom step of the escalator on the first floor. We analyzed time instead of distance to the escalator because time includes hesitations while participants were not moving. ‘Average Vertical Head Movement’ was measured as the difference (measured in degrees) in camera elevation angle between consecutive data points averaged over the analysis window. Cosine similarity was measured between the vector of a participant’s heading and the vector pointing to the first step on the escalator from the participant’s position. ‘Average Cosine Similarity’ was the mean of these values throughout the analysis window, ranging on a scale of ‘$-1$’ to ‘1’. Specifically, ‘1’ means that participants were heading directly towards the escalator, and ‘$-1$’ means that they were heading in the opposite direction. Irrespective of this difference in scale, ‘Average Cosine Similarity’ and angular differences produce the same pattern of results. A more detailed description and calculation of each wayfinding behavioral measure is provided in the “Supplementary materials” in the “Data analysis” section.

‘Destination visibility’ represented the extent to which the destination was visible from each point along the participants’ trajectories for each trial. It was calculated during post-processing by casting homogeneous rays to the destination’s surface (bounded by the field of view) and counting the percentage of rays reaching the destination. These visibility values ranged from 0 to 100%, where 0% was completely non-visible and 100% indicated that the destination filled the participants’ field of view without occlusions, see the “Supplementary materials” (“Data analysis” section) for more details. ‘Average Destination Visibility’ was calculated as the mean of these values from all recorded positions between the starting point and the escalator (i.e., throughout the analysis window). Furthermore, ‘Average Destination Visibility’ was used to segment the data into two ‘Visibility Conditions’, the ’non-visible’ (NV) condition with an ‘Average Destination Visibility’ equal to zero (406 trials) and the matching ’visible’ (V) condition (406 trials).

To test our hypotheses regarding the relationship between the visibility and wayfinding behavioral characteristics, we performed two types of analysis. First, we conducted regression analyses to determine the form of the relationships between ‘Average Destination Visibility’ and each of the wayfinding behavioral measures. Second, we combined the effects of ‘Visibility Condition’, atria-type, block, and session into a Linear Mixed Effects Regression (LMER) analysis^39^ for each of the three behavioral measures. LMER is an appropriate tool here because it allows us to combine several different factors. By including participants as a random factor, LMER also allows us to account for the variability attributable to individual participants and multiple measurements over blocks.

**Figure 2:**
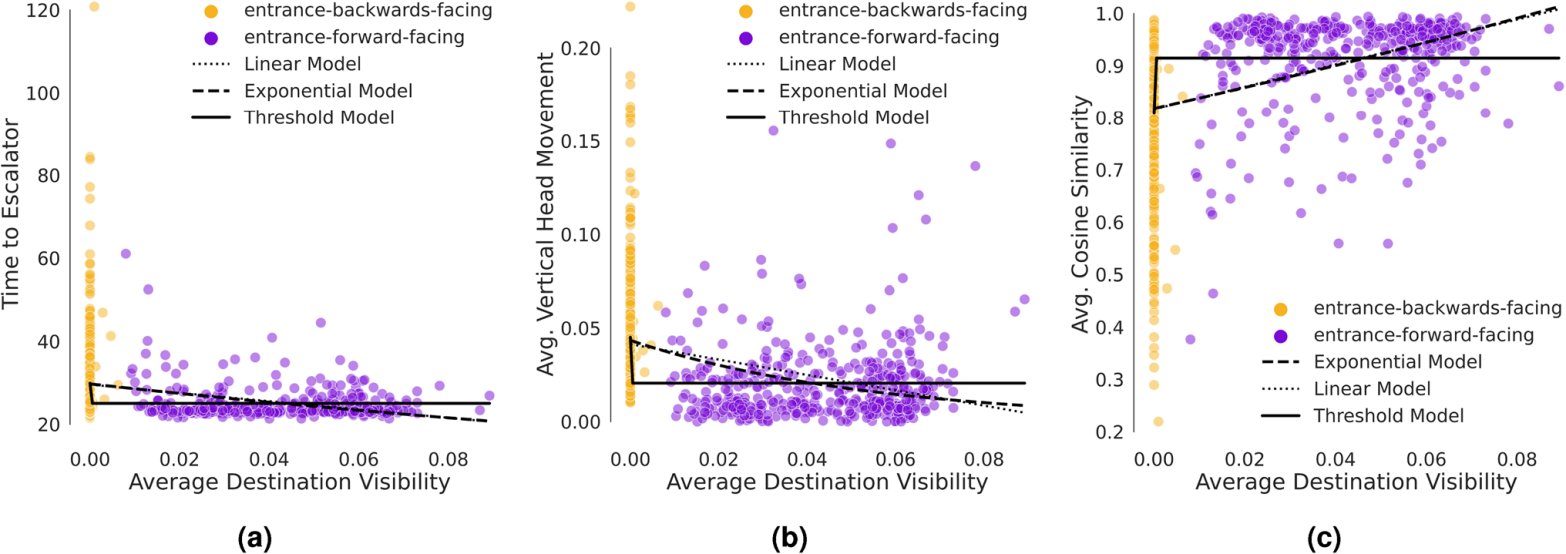
The raw data and fitted models showing the relationship between ‘Average Destination Visibility’ and behavioral wayfinding measures, (**a**) ‘Time to Escalator’, (**b**) ‘Average Vertical Head Movement’, (**c**) ‘Average Cosine Similarity’ between participants’ heading and the vector pointing towards the escalator. Software used to create these figures: Python^34^ (version 3.5.8), https://www.python.org/downloads/release/python-358/; Seaborn^35^ (version 0.11.1), https://seaborn.pydata.org/index.html; Matplotlib^36^ (version 3.3.2), https://matplotlib.org/3.3.2/users/installing.html.

We conjectured that the form of the relationship between ‘Average Destination Visibility’ and the three behavioral measures could be linear or nonlinear. To compare different possible forms of these relationships, we fit three different models to the data (i.e., linear: $f(x)=a \cdot x+b$; exponential: $f(x)=a \cdot e^{b \cdot x }$; threshold: $f(x)=a$ if $x=0$ and $f(x)=b$ if $x>0$; where *x* is ‘Average Destination Visibility’ and *f*(*x*) is the wayfinding measure). Figure 2 and Supplementary Table S1 present the results of our regression analyses on the relationship between ‘Average Destination Visibility’ and the three wayfinding behavioral measures. From the Information Criteria (IC) perspective, the threshold and exponential models fit relatively well. These analyses support the notion of a nonlinear relationship between ‘Average Destination Visibility’ and each of the three wayfinding measures. This pattern may need to be interpreted with caution because the differences between model fits are not always large, depending on which information criteria is considered. At the same time, it is important to note that the extrapolated behavioural measures for the linear and exponential models (i.e., beyond the current regime of the observed ‘Average Destination Visibility’) would become physically impossible (i.e.,‘Average Cosine Similarity’ would be greater than 1 and ‘Time to Escalator’ and ‘Vertical Head Movement’ would be below the physical constraints). These practical implications alongside the performance advantage of the threshold model with respect to the IC makes it a reasonable choice. All of the model fits, information criteria, and model comparisons are available in the “Supplementary materials”.

Two distinct behaviors are observed in the fitted threshold model. First, when the destination is in sight (i.e., ‘Average Destination Visibility’ $> 0$), there were decreases in ‘Time to Escalator’ and ‘Average Vertical Head Movement’, as well as an increase in ‘Average Cosine Similarity’. Second, once the destination was out of sight (i.e., ‘Average Destination Visibility’ = 0), there was the opposite pattern whereby ‘Time to Escalator’ and ‘Average Vertical Head Movement’ increased substantially and ‘Average Cosine Similarity’ decreased. Once the destination was visible (whether barely or highly visible), participants required less time to reach the escalator, looked upwards less, and moved more directly towards the escalator. This pattern suggests that participants intended to switch to the upper floor once the destination was initially visible. In contrast, when the destination remained out of sight, participants’ looked upwards more, indicating an ‘active visual search’ and moved less directly towards the escalator, thus taking longer to reach it.

**Figure 3:**
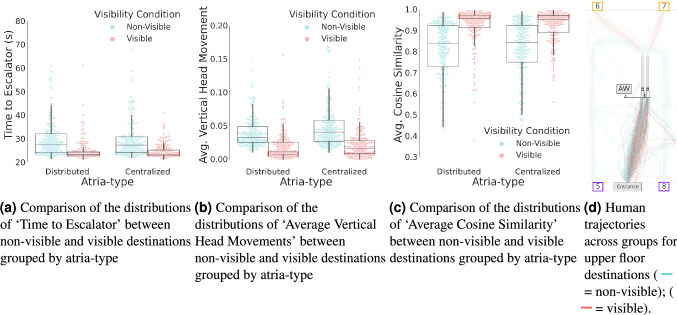
Figures (**a**–**c**) show the relationship between ‘visible’ or ‘non-visible’ trials (within each group, i.e., distributed versus centralized atria-type buildings) and each of the wayfinding behavioral measures. Figure (**d**) shows participants’ trajectories within the analysis window (across both groups), colored by visibility conditions. Software used to create these figures: Python^34^ (version 3.5.8), https://www.python.org/downloads/release/python-358/; Seaborn^35^ (version 0.11.1), https://seaborn.pydata.org/index.html; Matplotlib^36^ (version 3.3.2), https://matplotlib.org/3.3.2/users/installing.html; Rhino 6 for Windows (Version 6)^37^, https://www.rhino3d.com/download/.

To confirm the pattern of results suggested by the threshold model, we compared ‘Average Destination Visibility’ for different destinations on the second floor. ‘Average Destination Visibility’ for ‘entrance-backwards-facing’ destinations ranged from 0.000 to 0.006. Indeed, only 8 of these 414 trials had an ‘Average Destination Visibility’ greater than zero. In contrast,‘Average Destination Visibility’ for ‘entrance-forward-facing’ destinations ranged from 0.008 to 0.080 (see Fig. 2). This analysis shows that different destination locations were considerably dissimilar in terms of visibility, demonstrating non-overlapping distributions of ‘Average Destination Visibility’. This observed difference along with the threshold model provide the rational for our segmentation of trials into two ‘Visibility Conditions’. Figure 3 shows the relationship between ‘visible’ (V) and ‘non-visible’ (NV) trials within each group (i.e., distributed versus centralized buildings) and each of the wayfinding behavioral measures. In addition. Figure 3d shows participants’ trajectories within the analysis window colored by ‘Visibility Conditions’.

In the second analysis, we applied three LMERs to study participants’ wayfinding performance. We built one model for each of the three wayfinding behavior measures as a model response (i.e., ‘Time to Escalator’, ‘Average Vertical Head Movement’, and ‘Average Cosine Similarity’). Each model included fixed effects for ‘Visibility Condition’ (non-visible versus visible), atria-type (centralized versus distributed), block (first, second, and third), and session (Day 1 versus Day 2) as well as a random intercept for participants. We used participants as a random effect following conventions in cognitive science to account for repeated measures^40^. The LMER models followed this formula (in R Notation): $\text{ PerformanceMeasure } \sim \text{ AtriaType } + \text{ Block } + \text{ VisibilityCondition } + \text{ Session } + ( 1 | \text{ Participant})$. The results of this analysis are provided in Tables 1, 2, and 3, varying ‘$\text{ PerformanceMeasure }$’ for each of the three behavioral measures. The statistical significance threshold after Bonferroni^41^ correction for the desktop VR study (considering a total of 21 tests for the 2 hypotheses with an original $\alpha _{uncorrected}=0.05$) is set to $\alpha =0.001 (< 0.05/21)$).

**Table 1:** Results of the ‘Time to Escalator’ linear mixed effects model regression (LEMR), $\text{ TimeToEscalator } \sim \text{ AtriaType } + \text{ Block } + \text{ VisibilityCondition } + \text{ Session } + ( 1 | \text{ Participant})$, we denote p-values satisfying the Bonferroni corrected alpha with ***.

	Coef.	Std. Err.	z	p $> |{\text {z}}|$	[0.025	0.975]
Intercept	34.765	1.830	18.996	$$<\mathbf{0}.001 ***$$	31.178	38.352
Visibility condition (NV-V)	$$-4.935$$	0.445	$$-11.087$$	$$<\mathbf{0}.001 ***$$	$$-5.807$$	$$-4.062$$
Atria type	$-0.114$	1.053	$-0.108$	0.914	$-2.178$	1.951
Block	$$-2.095$$	0.272	$$-7.701$$	$$<\mathbf{0}.001 ***$$	$$-2.628$$	$$-1.562$$
Session	$-0.978$	1.053	$-0.928$	0.353	$-3.042$	1.087
Group var	15.727	0.543

**Table 2:** Results of the ‘Average Vertical Head Movement’ Linear mixed effects model regression (LEMR), $\text{ VerticalHeadMovement} \sim \text{ AtriaType } + \text{ Block } + \text{ VisibilityCondition } + \text{ Session } + ( 1 | \text{ Participant})$, we denote p-values satisfying the Bonferroni corrected alpha with ***.

	Coef.	Std. Err.	z	p $> |{\text {z}}|$	[0.025	0.975]
Intercept	0.041	0.006	6.641	<0.001 ***	0.029	0.052
Visibility condition (NV-V)	$-0.025$	0.002	$-15.520$	<0.001***	$-0.028$	$-0.022$
Atria Type	0.006	0.003	1.662	0.096	$-0.001$	0.013
Block	$-0.002$	0.001	$-2.363$	0.018	$-0.004$	$-0.000$
Session	0.001	0.003	0.340	0.734	$-0.006$	0.008
Group var	0.000	0.002

**Table 3:** Results of the‘Average Cosine Similarity’ Linear mixed effects model regression (LEMR), $\text{ AvgCosineSimilarity } \sim \text{ AtriaType } + \text{ Block } + \text{ VisibilityCondition } + \text{ Session } + ( 1 | \text{ Participant})$, we denote p-values satisfying the Bonferroni corrected alpha with ***.

	Coef.	Std. Err.	z	p $> |{\text {z}}|$	[0.025	0.975]
Intercept	0.723	0.031	23.107	<0.001 ***	0.661	0.784
Visibility condition (NV-V)	0.107	0.007	16.481	$$<\mathbf{0}.001***$$	0.095	0.120
Atria type	0.002	0.018	0.111	0.912	$-0.034$	0.038
Block	0.039	0.004	9.747	$$<\mathbf{0}.001***$$	0.031	0.047
Session	0.010	0.018	0.539	0.590	$-0.026$	0.046
Group var	0.005	0.011				

This LMER analysis provides confirmation of our hypothesis regarding the effect of ‘Visibility Condition’ on wayfinding performance. Specifically, the LMER analysis revealed that ‘Time to Escalator’ had a global intercept of 34.76 s (i.e., for the non-visible destinations during the first block). When the destinations were visible, participants took on average 4.935 s less. The LMER model shows that this effect is statistically significant, (Coef $=-4.935$, Std. Err. $=0.445$, z $=-11.087$, p $<0.001$, and 95% confidence interval $[-5.807, -4.062]$), see Table 1. Notably, replacing ‘Time to Escalator’ with a measure of distance to the escalator did not change the pattern of results. To provide further evidence regarding the significance of the ‘Visibility Condition’ effect, we provide a two-tailed (non-parametric) Wilcoxon signed-rank test in the “Supplementary materials”. The LMER analysis also revealed that ‘Average Vertical Head Movement’ had a global intercept of $0.041^{\circ }$. This measure was significantly lower (by $0.025^{\circ }$) when the destination was visible (Coef. $=-0.025$, Std. Err. $=0.002$, z $=-15.520^{\circ }$, p $<0.001$, and 95% confidence interval $[-0.028, -0.022]$), see Table 2. In addition, the LMER analysis revealed that the ‘Average Cosine Similarity’ had a global intercept of 0.723, which significantly increased by 0.107 when the destination was visible (Coef. = 0.107, Std. Err. = 0.007, z = 16.481, p $<0.001$, and 95% confidence interval [0.095, 0.120]), see Table 2.

Considering our hypothesis regarding the effect of the continuity of sight-lines on wayfinding performance, the effect of atria-type was not statistically significant for any of the LMER models representing each of the different response variables (‘Time to Escalator’: Coef $=-0.114$, Std. Err. = 1.053, z $=-0.108$, p = 0.914, and 95% confidence interval $[-2.178, 1.951]$), see Table 1, (‘Average Vertical Head Movement’: Coef = 0.006, Std. Err. = 0.003, z = 1.662, p = 0.096, and 95% confidence interval $[-0.001, 0.013]$), see Table 2, (‘Average Cosine Similarity’: Coef. = 0.002, Std. Err. = 0.018, z = 0.111, p = 0.912, and 95% confidence interval $[-0.034, 0.038]$), see Table 3. To further explore this null effect, we provide an additional analysis using a two-tailed Wilcoxon signed-rank tests in the “Supplementary materials”. Interestingly, this null result suggests that the fragmentation of sight lines by visual barriers does not affect the wayfinding behavioral characteristics, although it does affect ‘Average Destination Visibility’ of the destination (see Supplementary Fig. S2).

**Figure 4:**
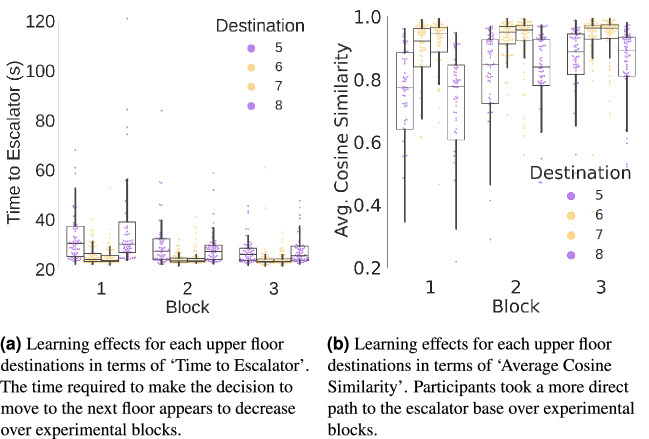
Learning effects over blocks of trials. Software used to create these figure: Python^34^ (version 3.5.8), https://www.python.org/downloads/release/python-358/; Seaborn^35^ (version 0.11.1), https://seaborn.pydata.org/index.html; Matplotlib^36^ (version 3.3.2), https://matplotlib.org/3.3.2/users/installing.html.

In addition, we tested for learning effects over blocks of trials for each destination. Because we randomized the locations of the destinations, any block effect represents learning of the task rather than spatial learning. Interestingly, this analysis reveals a significant learning effect across all trials with respect to ‘Time to Escalator’, (Coef $=-2.095$, Std. Err. = 0.272, z $=-7.701$, p $<0.001$, and 95% confidence interval $[-2.628, -1.562]$), see Table 1 and ‘Average Cosine Similarity’ (Coef. = 0.039, Std.Err. = 0.004, z = 9.747, p = $<0.001$, and 95% confidence interval [0.031, 0.047]), see Table 3. This finding suggests that participants walked 2.095 s less on average to the escalator for each new block (see Fig. 4a) and that participants increased their ’Average Cosine Similarity’ by 0.039 for each new block (see Fig. 4b). However, this effect was not present in the ‘Average Vertical Head Movement’ measure (Coef $=-0.002$, Std. Err. = 0.001, z $=-2.363$, p = 0.018, and 95% confidence interval $[-0.004, 0.000]$), see Table 2.

Finally, given that the experiment was conducted in two sessions with different groups of participants, we checked whether these two sessions had any significant effect on the wayfinding behavioral measures. Here, we expected and confirmed null effects for all of the behavioral measures (‘Time to Escalator’: Coef $=-0.978$, Std. Err. = 1.053, z $=-0.928$, p = 0.353, and 95% confidence interval $[-3.042, 1.087]$) , see Table 1, (‘Average Vertical Head Movement’: Coef = 0.001, Std.Err. = 0.003, z = 0.340, p = 0.734, and 95% confidence interval $[-0.006, 0.008]$), see Table 2, (‘Average Cosine Similarity’: Coef. = 0.010, Std. Err. = 0.018, z = 0.539, p = 0.590, and 95% confidence interval $[-0.026, 0.046]$), see Table 3.

### Study 2: Humans’ versus agents’ wayfinding performance

To demonstrate the application of the wayfinding behavioral patterns identified in the desktop VR study (“Study 1: Human wayfinding behavior in multilevel virtual environments”), we implemented a visibility-based cognitive agent model. The visibility-based cognitive agent model encompasses the behavioral mechanism observed in the desktop VR study along with other reported mechanisms impacting wayfinding behavior (i.e., a measure of isovist drift; see Supplementary Fig. S5). In addition, for comparison, we created a second agent model that was driven by the shortest path algorithm A*^42^ which is often used in the field of pedestrian modeling and represents an optimal search behavior that assumes global knowledge of the navigation environment^43^). We used the observations of human wayfinding from the desktop VR study as our benchmark to which both shortest-path agents’ and visibility-based cognitive agents’ performances can be compared to. To compare the performance of both agent types to observed human behavior, we replicated the desktop VR experimental setup and conducted two Monte Carlo type simulation experiments (one with visibility-based cognitive agents and one with shortest-path agents).

**Figure 5:**
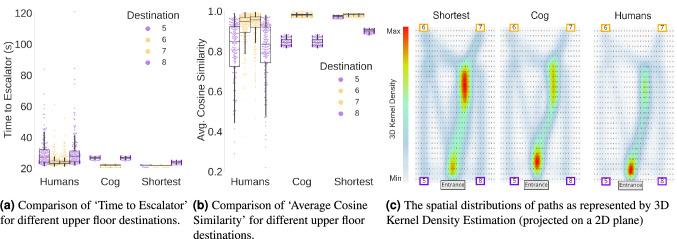
A comparison of wayfinding behavioral characteristics and path distributions across agent types and compared to observed human behavior. Software used to create these figures: Python^34^ (version 3.5.8), https://www.python.org/downloads/release/python-358/; Seaborn^35^ (version 0.11.1), https://seaborn.pydata.org/index.html; Matplotlib^36^ (version 3.3.2), https://matplotlib.org/3.3.2/users/installing.html; QGis^38^ (version 3.16), https://qgis.org/en/site/forusers/download.html.

The wayfinding behavior of the visibility-based cognitive agents and the shortest-path agents were compared to the human participants’ behaviors for three measures, ‘Time to Escalator’, ‘Average Cosine Similarity’, and ‘Spatial Distribution of Paths’ (represented by a Kernel Density Estimation). Figure 5a,b show the comparison between agents and human behavior across these three measures for upper floor destinations. As expected, the patterns of ‘Time to Escalator’ and ‘Average Cosine Similarity’ that were observed in humans were successfully captured by the visibility-based cognitive agents, while the shortest-path agents were unable to replicate the same patterns. This suggests that the visibility-based cognitive agents, similarly to humans, reached the escalator faster for the case of ‘entrance-forward-facing’ destinations compared to ‘entrance-backwards-facing’ destinations. However, unlike the human participants, ‘Time to Escalator’ for the shortest-path agents was invariant to the location of the destinations and its ‘Average Destination Visibility’. Similarly, for the case of ‘Average Cosine Similarity’, the visibility-based cognitive agents replicated human behavior by moving more directly towards the staircase while wayfinding towards ‘entrance-forward-facing’ destinations compared to wayfinding towards ‘entrance-backwards-facing’ destinations. In contrast, shortest-path agents were agnostic to the ‘Average Destination Visibility’ of the destinations and thus were not able to replicate either of these wayfinding behavioral patterns. Despite the observed similarity between cognitive agents and human behavior for the first two measures, a comparison of agents and humans for the third measure of ‘Spatial Distribution of Paths’ using Gaussian Kernel Density Estimation (KDE) shows a marked difference between cognitive agents and humans (see Fig. 5c).

**Figure 6:**
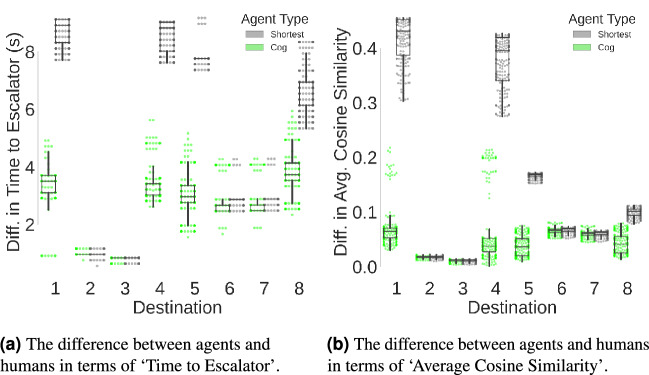
Differences between agents and humans in terms of three wayfinding behavioral metrics. Software used to create this figure: Python^34^ (version 3.5.8), https://www.python.org/downloads/release/python-358/; Seaborn^35^ (version 0.11.1), https://seaborn.pydata.org/index.html; Matplotlib^36^ (version 3.3.2), https://matplotlib.org/3.3.2/users/installing.html.

To quantify the similarity, or ‘goodness of fit’^44^, between the agent models and observed human wayfinding behavior across measures, the $L^1 Norm$ of the differences (i.e. sum of absolute differences) in ‘Time to Escalator’ and ‘Average Cosine Similarity’ measures were calculated (see Fig. 6a,b, respectively). To analyze these differences between human participants and the two agent-types we apply two LMERs. We build one model for each of the differences in behavioral measures (i.e., ‘Difference in Time to Escalator’ and ‘Difference in Average Cosine Similarity’) as model responses. Each model included the agent-type (cognitive agent versus shortest-path agent) as a fixed effect and a random intercept for agents IDs. The LMER models followed this formula (in R Notation): $\text{ DifferenceInMeasure } \sim \text{ AgentType } + ( 1 | \text{ Agent})$. The statistical significance threshold for the analysis of agents’ behavior after Bonferroni correction (considering a total of 7 tests with an original $\alpha _{uncorrected}=0.05$ is set to $\alpha =0.001 (< 0.05/7)$. The LMER analysis revealed a statistically significant effect of the agent-type (cognitive agent versus shortest-path agent) resulting in an increase of 2.319 s in ‘Difference in Time to Escalator’, (Coef = 2.319, Std.Err. = 0.045, z = 51.147, p $<0.001$, and 95% confidence interval [2.231, 2.408]), see Table 4. This means that the difference in time taken to reach the escalator for the case of shortest-path agents was significantly larger (2.319 s) than that demonstrated by cognitive agents. Moreover, the LMER analysis also showed a statistically significant effect of the agent-type (cognitive agent versus shortest-path agent) resulting in an increase of 0.106 in the ‘Difference in Average Cosine Similarity’, (Coef = 0.106, Std. Err. = 0.003, z = 41.223, p $<0.001$, and 95% confidence interval [0.101, 0.111]), see Table 5. This means that the difference in directness towards the bottom of the escalator for the case of shortest-path agents was significantly (0.106) larger than that demonstrated by cognitive agents. An additional non-parametric analysis (using a Wilcoxon signed-rank test) of the differences between agents’ and humans’ wayfinding is provided in the “Supplementary materials” alongside a more fine-grained analysis of spatial differences using Dynamic Time Warping (DTW). Notably, neither of the agents were subjected to any parameter tuning or training because the aim of this study was to demonstrate the ‘behavioral first principles’ extracted from the desktop VR study rather than achieve the greatest model fit. The application of these agents would require a tuning process and rigorous validation.

**Table 4:** Results of the ‘Difference in Time to Escalator’ Mixed linear model regression, $\text{ diffTime } \sim \text{ AgentType } + ( 1 | \text{ AgentID})$, we denote p-values satisfying the Bonferroni corrected alpha with ***.

	Coef.	Std. Err.	z	p $> |{\text {z}}|$	[0.025	0.975]
Intercept	2.600	0.524	4.958	$$<\mathbf{0}.001***$$	1.572	3.627
Agent type (to shortest)	2.319	0.045	51.147	$$<\mathbf{0}.001***$$	2.231	2.408
Group var	4.383	1.250

**Table 5:** Results of the ‘Difference in Average Cosine Similarity’ mixed linear model regression, $\text{ diffCos } \sim \text{ AgentType } + ( 1 | \text{ Agent})$, we denote p-values satisfying the Bonferroni corrected alpha with ***.

	Coef.	Std. Err.	z	p $> |{\text {z}}|$	[0.025	0.975]
Intercept	0.046	0.021	2.181	0.029	0.005	0.087
Type to shortest	0.106	0.003	41.223	$$<\mathbf{0}.001 ***$$	0.101	0.111
Group var	0.007	0.036

## Discussion

The main purpose of the present studies was to understand and model the manner in which visibility affects wayfinding behavior towards novel destinations in unfamiliar and multilevel environments. We expected that the visibility of destinations as well as the continuity of sight-lines along the vertical dimension would affect various wayfinding behavioral measures and performance. To test this hypothesis, we conducted a desktop VR experiment in which two aspects of visibility were manipulated: (1) each destination’s location with respect to visibility from the entrance and (2) the environmental geometry of the atria that visibly connected floors along the vertical dimension (i.e., atria-type, distributed versus centralized). To model the wayfinding behavioral pattern emerging from our findings, we conducted simulation experiments and developed a vision-based cognitive agent that was able to better replicate the observed human behavior when compared to a shortest-path agent.

Our first hypothesis for the desktop VR experiment was largely confirmed because the visibility of different destinations (i.e., ‘Average Destination Visibility’) significantly affected wayfinding behavior during between-floor trials (i.e., within-group) regardless of the atria-type manipulation (i.e., between-groups). Specifically, we observed two distinct behaviors for between-floor trials. When the destination was not visible, there was an increase in ‘Average Vertical Head Movement’, a reduced ‘Average Cosine Similarity’ between the heading and the optimal direction, and consequently, an increase in time and distance to the escalator. In contrast, when the destination was visible, we observed a reduced ‘Average Vertical Head Movement’, an increased ‘Average Cosine Similarity’ between the heading and the optimal direction, and consequently, a decrease in time and distance to the escalator. These results suggest that, once the destination was in sight, participants decided to switch floors and move up towards the destination regardless of the extent to which it was visible. Otherwise, participants performed a ‘visual search’. A possible interpretation of these findings is that it may represent two distinct wayfinding behaviors, ‘exploration’ versus ‘exploitation’, that can be predicted using ‘Average Destination Visibility’. While previous studies have concluded that ‘more visibility’ corresponds with ‘more certainty’ and ‘less risk’^11^, we found that the degree of visibility (i.e., high versus low) played a minor role (if any). The nonlinear relationship between ‘Average Destination Visibility’ and wayfinding behavioral measures is the key contribution of our paper. Although it is unexpected and counterintuitive, it highlights the critical role visibility plays in how humans manage the trade-off between exploration and exploitation during wayfinding in the vertical.

Furthermore, our results demonstrate a consistent improvement in wayfinding performance for the same ‘entrance-forward-facing’ or ‘entrance-backwards-facing’ destinations as the experiment was repeated across blocks of trials. This secondary finding shows a significant effect of learning on wayfinding behavior, despite the randomization of destination locations across trials (see “Methods” section for more details). Nonetheless, while participants’ search behavior became more efficient over blocks, the relationship between their wayfinding performance and ‘Average Destination Visibility’ was independent of the learning effect. This means that the significant effect of ‘Average Destination Visibility’ was consistent over blocks.

Although environmental geometry did change the continuity of sight-lines for destinations on the upper floor, this manipulation of visibility did not significantly affect wayfinding behavior, rejecting our second hypothesis. This null result suggests that the fragmented visibility introduced by the distributed atria was not sufficient to reduce the efficiency with which participants reached the escalator on their way towards the destination. Indeed, once participants had the destination on the upper floor in sight, they moved efficiently towards the escalator. This finding appears to contradict previous research on visibility and indoor wayfinding that has suggested that fragmented visibility can affect wayfinding behavior^31^. However, He et al. compared a naturalistic condition in which visibility was intermittently obstructed by obstacles with an X-ray condition in which these obstacles were translucent. In contrast, the present study involved a manipulation of the continuity of sight-lines by changing the atria-type between groups such that the visibility of the destination was different between groups. Notably, the objective of He et al. to enhance navigation through virtual environments was different from the objective of the present study, but the potential interaction between the amount of visibility and the continuity of visibility may be explored in future research to inform the design of real environments with wayfinding in mind.

Based on our findings from the desktop VR study, we also developed visibility-based cognitive agents. In a series of simulation experiments, we compared visibility-based cognitive agents to agents with complete knowledge and without visibility (i.e., shortest path agents). The virtual environments and wayfinding task were the same as during the desktop VR experiment, so that we could assess the similarity of agents’ wayfinding behavior to humans’ wayfinding behavior. Indeed, our modeling of visibility as a three-dimensional field of view alongside the application of the ‘isovist drift measure’ allowed us to reproduce the relationship between ‘Average Destination Visibility’ and wayfinding behavioral measures and may thus be used to constrain the prediction of wayfinding in unfamiliar, multilevel environments. Drift is the distance in meters between the location from which the isovist was cast and its ‘center of gravity’^11^. Our results extend previous research where isovist drift measures have been applied to model exploration behavior^45,46^, suggesting that isovist characteristics can be used, to some extent, to simulate wayfinding movement. However, we also found that the spatial distributions of the trajectories from humans and cognitive agents were markedly different. This finding is largely attributable to the similarity of trajectories once the destination was visible. While humans appeared to switch to a ‘destination approach’ mode, the visibility of the destination along the path continued to affect the wayfinding of the cognitive agents.

### Limitations and future directions

Our novel findings regarding visibility and wayfinding must be considered alongside the limitations of our approach. In principle, our experimental design does not allow us to draw inferences concerning the mental representation of the multilevel environments. There are many open questions regarding, for example, vertical versus horizontal anisotropy^5,20,21^ and the representation of hierarchical environments^12,22–26^ that are related to our study but cannot be addressed due to our choice of task structure and the use of desktop VR.

Our task involved wayfinding towards unfamiliar destinations and thus did not test participants’ mental representations during wayfinding in the vertical which could inform their selection of wayfinding strategies^12,22–26^. In future work, researchers can compare participants’ performance in similar visibility tasks before and after learning an environment in order to test whether memory would inform strategy selection. This could be further complemented by a more in-depth understanding of participants’ spatial strategies using questionnaires such as Lawton indoor wayfinding scale^47^.

With regards to desktop VR, movement using visual motion does not provide users with idiothetic cues that can form the basis of the spatial representation of large 3D environments^21^. Although desktop VR has been used to elicit realistic trajectories in locomotion^33^ and evacuation tasks^48^, it is possible that participants in the present study would have searched for destinations in a different manner if, for example, their view of the environment was not attached to an avatar that they moved with their hands via the control interface. Future work can directly test this possibility by measuring head movements and bodily trajectories in an ambulatory VR setup.

Finally, the visibility-based cognitive agent lacks a more comprehensive model of memory, following recent state-of-the-art research in artificial intelligence that was inspired by findings on grid cells to simulate navigation^49^. Future work could combine the latter with our own findings concerning the effects of visibility on wayfinding in the vertical and, specifically, the discrete transition between exploration and exploitation as a function of visibility. Such a holistic approach could provide a novel testing ground for scientific experiments and theoretical inquiries that could improve our understanding of the interplay between complex environments and human cognition.

## Methods

### Study 1: A desktop VR experiment

#### Participants

Two experimental sessions took place during October 2019 at ETH Zurich’s Decision Science Laboratory (DeSciL). In total, 69 participants (44 females and 25 males; mean age = 23.6 years; SD = 3.16; age range = 18–37) were recruited via the University of Zurich’s Registration Center for Study Participants (https://www.uast.uzh.ch). The study was approved by the Research Ethics Committee of ETH Zurich (2015-N-37). All methods and experiments described in this section were performed in accordance with the relevant guidelines and regulations. The main inclusion criteria were normal or corrected-to-normal vision and the ability to discriminate colors (i.e., colorblind individuals were excluded). All participants completed an informed consent form before the study. Participants required, on average, 20.14 min (completion time range = 17.33–29.73 minutes) to complete the experiment and were paid 25–35 CHF (mean payment = 30CHF), depending on their performance.

#### Materials

Similarly to^33^, participants were seated in a room with 36 cubicles, each of which contains a desktop computer used in the experiment. The study materials included two virtual models of 3D multilevel environments generated specifically for this experiment. The first floors of both environments were identical, consisting of enclosed rooms situated along the perimeter of a rectangular shaped floor (see Fig. 1). The rooms had glass doors, enabling participants to see through the door inside of each room. The entrance to the first floor was from one of the shorter sides of the rectangle, leading to a central open area (i.e., barrier free) with a single staircase leading upwards.

Four possible destinations were located on the first floor, two of which were ‘entrance-forward-facing’ and the other two of which were ‘entrance-backwards-facing’. The second floor surface included an atria with either a centralized pattern (i.e., a single atrium; total area = 288 m$^{2}$) or a distributed pattern (i.e., three atria; total area = 288 m$^{2}$). On the second floor, another set of four possible destinations was positioned at the corners of each floor. Two of these destinations were ‘entrance-forward-facing’, and the other two destinations were ‘entrance-backwards-facing’ relative to participants’ initial positions and headings, which were fixed across trials and groups. The decision to include first-floor destinations intended to ensure that participants would not automatically expect each destination to be on the second floor. Otherwise, it we would not have been possible to infer when participants decided to switch floors. To control for the amount of walkable space on the second floor across both groups (i.e. distributed versus centralized atria), a fence restricting movement to the perimeter of the space was placed. This ensured that the paths were still comparable between groups, despite the atria variation.

Participants’ starting locations across both groups and trials were fixed and set to the main entrance on the first floor. Destinations were set with respect to participants’ initial heading. Destination doors varied with respect to color, and this color was randomized independent of its location for each trial. It is important to note that our use of color to indicate the destinations produces beacon instead of associative cues^50^. Whereas associative landmarks would lead to much more enduring route knowledge than beacon landmarks, in the present study, we purposefully controlled the extent to which participants could have learned specific routes by randomly switching the colors of potential targets from trial to trial in order to study the specific effects of visibility on wayfinding.

**Figure 7:**
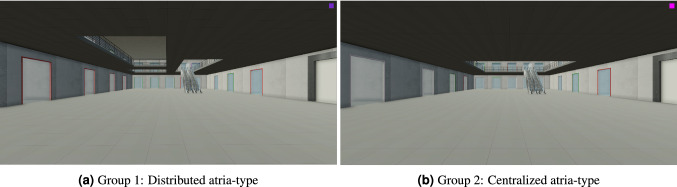
Exemplary screenshots from the desktop VR study (captured from the Unity3D^52^ game engine) showing a first-person perspective taken from the entrance (starting point) for each of the two atria-types. The overall area of the second floor in each group was the same for both the distributed and centralized atria (i.e., 288 m$^{2}$). Software used to create this figure: Unity3D^52^ (version 2018.4.16f1), https://unity3d.com/unity/whats-new/2018.4.16f1.

The experimental software and processing of the virtual environment was developed using Unity3D game engine (Unity Technologies). Similarly to^51^, Unity3D was used to support rendering, character control, and movement recording, as well as to replay participants’ movement paths in the virtual environment. We recorded participants’ trajectories by logging their positions and orientations every frame (i.e., approximately every 0.02 s). Each participant had a first-person view of the navigation environment and could navigate using a keyboard and mouse (See Fig. 7). Movements and rotations were described according to the conventions of Unity3D, the game engine used to design and implement the experiment. Accordingly, navigation included the following translations and rotations: forward/backward translations, left/right translations, up/down translations, left/right rotations (i.e., yaw), and up/down rotations (i.e., pitch). To simplify the control setup, a homogeneous virtual character was assumed as reported in^33^ (height = 1.8 m; eye height = 1.7 m; shoulder width = 0.25 m; maximum forward walking speed = 1.3 m/s; backwards and lateral moving speed = 0.6 m/s).

#### Procedure

Random assignment was applied to split participants between groups (i.e., atria-type: distributed versus centralized). Each computer in the 36-seat laboratory was preselected for a specific atria-type. As participants entered the laboratory, they were given a random card indicating their seat. These cards were shuffled by an experimenter before the participants arrived.

Participants could not see the screen of the participants seated in other cubicles and were instructed not to communicate with each other during the experiment. Each experimental session started with a 5–10 min training phase, during which participants learned how to navigate in the virtual environment. This training phase was designed to review all possible movements, including movement between floors by means of stairs. The variability in the duration of the training session was due to participants being allowed to take breaks during training (but not during the later experimental trials). The environment used for training was a different building to avoid any spatial learning or transfer between training and testing environments, see exemplary screenshots of the training environment in the “Supplementary materials”, Fig. S3.

Testing was composed of three blocks, each of which consisted of eight trials. The order of trials (each with a different destination) was randomized within each block. During each trial, participants were asked to search for a doorway of a particular color. Once participants arrived at the destination, they automatically continued to the next trial.

#### Design

There were two independent variables of interest: (1) the location of destinations with respect to its visibility from the starting point at each trial (i.e.,‘entrance-forward facing’ or ‘entrance-backwards-facing’) and (2) the atria-type (distributed versus centralized atria), resulting in fragmented or continuous visibility towards the destinations. In our analysis, we also considered block (first, second, and third) and session (Day 1 versus Day 2).

Three dependent wayfinding behavioral measures are used to test our hypotheses: (1)‘Time to Escalator’, (2) ‘Average Vertical Head Movement’, and (3) ‘Average Cosine Similarity’. ‘Time to Escalator’ is defined as the time required to reach the bottom step of the escalator on the first floor, referred to as the analysis window. ‘Average Vertical Head Movement’ is measured as the difference in camera elevation between consecutive measures averaged over the analysis window. Cosine similarity is measured as the cosine of the angle between the vector of a participants’ heading, and the vector pointing to the first step on the escalator from their position. ‘Average Cosine Similarity’ is the average of these values during the analysis window.

Two hypotheses were set:

- A higher degree of visibility towards upper floor destinations would have a positive and significant effect on wayfinding behavioral measures (within-group).
- Continuous visibility in the case of the centralized atria would have a significant effect on wayfinding behavioral characteristics compared to fragmented visibility in the case of the distributed atria (between-group).

To test these hypotheses, we conducted Linear Mixed Effects Regression (LMER) for each of the three dependent measures using Python’s statsmodels library. Each of these models included fixed effects for ‘Visibility Condition’ (non-visible versus visible), atria-type (centralized versus distributed), block (first, second, and third), and session (Day 1 versus Day 2), as well as a random intercept for participants. The LMER models follow this formula (in R Notation): $\text{ PerformanceMeasure } \sim \text{ AtriaType } + \text{ Block } + \text{ VisibilityCondition } + \text{ Session } + ( 1 | \text{ Participant})$.

### Study 2: Visibility-based cognitive agents

#### The visibility-based cognitive agent model

Our proposed visibility-based cognitive agent integrates key findings from the desktop VR study to perform goal-directed wayfinding in an unfamiliar and multilevel environment. Agents’ geometry and walking characteristics correspond to that of the First Person Controller (FPC) used by human participants to navigate within the virtual environment (height = 1.8 m; eye height = 1.7 m; shoulder width = 0.25 m; maximum forward walking speed = 1.3 m/s; backwards and lateral moving speed = 0.6 m/s).

Agents’ movements were constrained by the same continuous three-dimensional environment set in the desktop VR experiment, but the agents’ environmental perception and decision making were aided by additional layers of information. The first layer divided the environment into multiple arrays of rectangular grid cells of different resolutions. A second, higher-order layer of the environment was zones. Zones divide the walkable environment into three-dimensional convex volumes. Zone boundaries are determined by bounding surfaces such as walls, floors, ceiling, and doors. An agent was able to visually perceive the environment by means of a three-dimensional field of view, originating from eye-level height and calculated in real time. Using its field of view, the agent captured the distribution of barriers that occluded sight-lines. The information captured by the agent was used to perform transitions between wayfinding states, as described in the finite-state machine (FSM) diagram in Supplementary Fig. S5.

To transition from the initial state at which agents’ are spawned, agents analyzed visible information to check if the destination was in-sight or out-of-sight (i.e., within or outside of their three-dimensional field of view). Accordingly, a transition was triggered to one of two wayfinding states: ‘Move to Escalator and Switch Floor’ (if the destination was in-sight) or ‘Explore’ (if the destination was not yet visible, i.e., out-of-sight). To avoid ‘looping’ behavior, visited zones (stored in a short-term memory object) could not be traversed more than once. This simple logic was based on a direct translation of the observed nonlinear relationship between ‘Average Destination Visibility’ and wayfinding behavior from the desktop VR study.

While in the ‘Move to Escalator and Switch Floor’ state, we used an A* algorithm to plan the shortest path towards the base of the escalator, considered as the intermediate target used to switch floor. While in the ‘Explore’ state, a spatial metric was used to guide the direction of agents’ exploration within the floor. This spatial metric, termed ’drift’, was calculated from a 2D isovist (horizontal angle = $360^{\circ }$; view range = 100 m) and cast from each node in the navigation graph. Drift was the distance in meters between the location from which the isovist was cast and its ‘center of gravity’^11^. Drift will tend towards a minimum value at the centers of spaces and along the center-lines of roads.

Previous research has shown that locations with minimum drift values have been correlated with the locations at which humans make wayfinding decisions, reflected in longer stay duration and directional changes. Similar drift measures have been applied previously to model exploration behavior^45^. Specifically^46^ compared aggregated movement patterns of pedestrians in downtown Santiago, Chile with behavior of three types of vision-based agents; ones that were moving randomly, ones that were moving toward their drift, and ones that were moving towards their longest line of sight. The main results show that drift-based agents were better suited to predict aggregated patterns than random behavior. This suggests that isovist characteristics can be used, to some extent, to simulate exploration movement. Accordingly, we generated an isovist from each cell in the navigation environment.

Isovists were cast from a eye height of 1.7 m, reflecting the average eye-height of an adult. Drift was calculated for each isovist polygon. An ‘isovist field’ storing the value of drift for each cell in the navigation environment was pre-computed. Agents were able to perceive the drift values of cells captured within their field of view (horizontal view angle = $150^{\circ }$; view range = 100 m) and calculated at each step. To explore the space, agents’ moved towards the cell with the minimal drift value. This process was intended to mimic the exploration-like movement that is informed by geometrical properties of visibility derived from the environmental configuration. By moving to the minimal drift point, agents reached the center lines of the environment at which they could perceive more information. This process iterated until the destination was in-sight or the floor had been ‘sufficiently explored’, which triggered the previously described state (‘Move to Escalator and Switch Floor’). Upon reaching the base of the escalator, and if the destination was visible, a third state ‘Move To Destination’ was triggered and a shortest path towards the destination was calculated. Otherwise, the agent remained in the ‘Explore’ state and continued to follow the minimal drift cells in the second floor until the destination was in-sight, see the “Supplementary materials” for details (Supplementary Fig. S5).

#### Simulation experiments

To compare agents’ with humans’ wayfinding performance (see Study 1), we replicated the desktop VR experiment setup and conducted two Monte-Carlo-type simulation experiments: (1) with visibility-based cognitive agents and (2) agents without visibility that rely solely on a ‘direct routing’ algorithm (i.e., the A* algorithm) to calculate the shortest path from the entrance to each destination. For each simulation experiment, a total of 1600 Monte-Carlo-type samples were taken. The agent’s initial heading was used as the random variable. The experimental framework used to develop and execute the simulation processing was based on the Unity3D game engine (Unity Technologies). We used Unity3D to render, control, record, and replay agents’ movements in the virtual environment. Trajectories were recorded by logging agents’ positions and orientations every 0.2 s. The simulations were executed using ETH Zurich’s Euler computing cluster through singularity-based containerization. To compare agents’ and humans’ wayfinding behaviors, two of the wayfinding behavioral measures used to quantify human behavior were also calculated for agents: (1)‘Time to Escalator’ and (2) ‘Average Cosine Similarity’.

## Supplementary Information

Supplementary Information 1.

## Data Availability

The wayfinding dataset obtained in the virtual reality experiment as well as data generated as part of the agent simulation is available in GitHub (https://github.com/MichalGath/WayfindingMultilevelEnvironments.git) The simulation code is available upon request. Figures in the manuscript and supplementary materials were created using the following software: Python^34^, (version 3.5.8) (using Seaborn^35^, (version 0.11.1) and Matplotlib^36^ (version 3.3.2) was used for Figs. 1, 2, 3a–c, 4, 5a,b, 6, S2, S4. Rhino 6 for Windows (Version 6)^37^ was used for panels 1, 2 and 3 in Figs. 1, 3d and for Figs. S7–S10 in the “Supplementary materials”. QGis^38^, (version 3.16) was used for panel 4 in Fig. 1 and for Fig. 5c. Unity3D^52^, (version 2018.4.16f1) game engine was used to capture the screenshots appearing in Fig. 7 and in Fig. S3. R^53^, (version 3.6.3 2020-02-29) using simr^54^ was used for Fig. S1. Latex (version TexLiveVersion 2020, Overaleaf), using dirtree, was used for Fig. S6. Miro (2021)^55^ was used for Fig. S5. The analysis files are available upon request.
