# Publisher Correction: Visibility matters during wayfinding in the vertical

**DOI:** 10.1038/s41598-021-99770-3

**Published:** 2021-10-06

**Authors:** Michal Gath-Morad, Tyler Thrash, Julia Schicker, Christoph Hölscher, Dirk Helbing, Leonel Enrique Aguilar Melgar

**Affiliations:** 1 Chair of Cognitive Science, ETH Zurich, Zurich, Switzerland; 2 Computational Social Science, ETH Zurich, Zurich, Switzerland; 3 Data Science, Systems and Services Laboratory, ETH Zurich, Zurich, Switzerland; 4 Department of Biology, Miami University, Oxford, USA

Correction to: *Scientific Reports* 10.1038/s41598-021-98439-1, published online 23 September 2021

The original version of this Article contained errors in the Affiliations.

Michal Gath-Morad, Tyler Thrash and Christoph Hölscher were incorrectly affiliated with ‘Computational Social Science, ETH Zurich, Zurich, Switzerland.’

The correct affiliation for Michal Gath-Morad and Christoph Hölscher is listed below.

Chair of Cognitive Science, ETH Zurich, Zurich, Switzerland.

The correct affiliations for Tyler Thrash are listed below.

Chair of Cognitive Science, ETH Zurich, Zurich, Switzerland.

Department of Biology, Miami University, Oxford, USA.

As a result, the Affiliations have been renumbered.

The original Article and accompanying Supplementary Information file have been corrected.

