# Supplementary material for: Visibility matters during wayfinding in the vertical
Source: Sci Rep. 2021 Sep 23;11:18980. doi: 10.1038/s41598-021-98439-1 (PMC8460814; doi:10.1038/s41598-021-98439-1)
Supplement: Supplementary file 1 — Supplementary Information 1. [file 41598_2021_98439_MOESM1_ESM.pdf]

Supplementary Materials

Visibility Matters During Wayfinding in the Vertical

Michal Gath-Morad[1,*], Tyler Thrash[1+4], Julia Schicker[1+2], Christoph Hölscher[1], Dirk Helbing[2], and Leonel Enrique Aguilar Melgar[1+2+3]

[1]Chair of Cognitive Science, ETH Zürich, Switzerland
[2]Computational Social Science, ETH Zürich, Switzerland
[3]Data Science, Systems and Services Laboratory, ETH Zurich, Switzerland
[4]Department of Biology, Miami University, United States
[*]corresponding author: michal.gath@gess.ethz.ch

A Study 1: desktop VR study

A.1 Power analysis for study 1

We performed two power analyses, an a priori power analysis that uses estimates and expected values for the experiment and an a posteriori power analysis that evaluates the actual power of the experiment. In these analyses, we focus on the effect of group (i.e., atria-type) on 'Time to Escalator'. We use 'Time to Escalator' as a proxy of the transition between exploration and exploitation behavior. 'Time to Escalator' is also simpler to provide reasonable educated guesses on its values for the a priori power analysis.

A.1.1 A priori power analysis

We built a prior Linear Mixed Effects Model (LME) of our experimental design. We assume participants' average time to reach the escalator to be 35 seconds with a standard deviation of 4 seconds (Random Effect). We then set our minimum effect of interest to be 1 second for each fixed effect. Finally, the residual standard deviation was set to 4 seconds. The LMER model includes the fixed effects of the 'Visibility Condition' (non-visible versus visible), atria-type (centralized versus distributed), block (first, second, and third), and included a random intercept for participants. Specifically, the LMER Model follows the following formula in R format: $\text{TimeToEscalator} \sim \text{AtriaType} + \text{Block} + \text{VisibilityCondition} + (1|\text{Participant})$. The model is built using R and the following libraries: lme4, for creating the model from scratch and simulating the response of the assumed model; EMAtools, for the calculation of Cohen's d; simr, to calculate the power of a model through simulation. This model yields an average simulated Cohen's d score of 0.25 for the group effect (1000 simulations of model responses).

We vary the number of participants and plot the power curve with 1000 simulations for each power calculation in Figure S1.

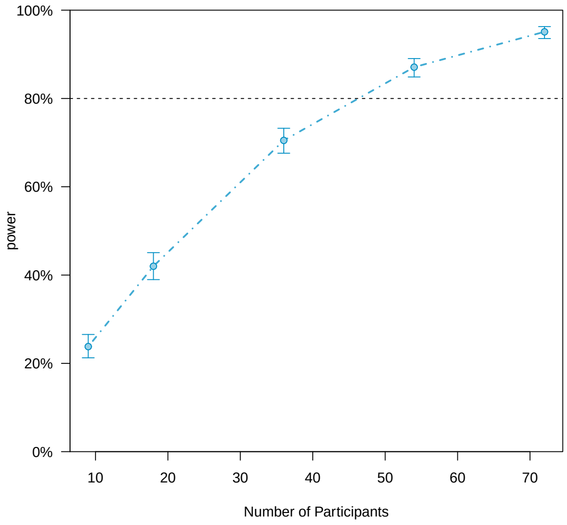

Figure S1. Power as a function of sample size according to the a priori power analysis for the desktop VR study. Software used to create this figure: R[1] (version 3.6.3 2020-02-29) using simr[2], https://cran.r-project.org/bin/windows/base/old/3.6.3/

From this analysis, we can conclude that more than 36 participants were needed (i.e., the capacity per session of our experimental lab facility). Hence, we required at least two sessions to achieve the desired statistical power. Thus, our proposed sample size of 72 participants is more than adequate for the main objective of this study and should also allow for moderate attrition (<10%).

A.1.2 A posteriori power analysis of the null result (atria-type)

In our posteriori power analysis, we evaluate the statistical power we achieved for our null effect of atria-type with the experimental sample size attained. We use the desktop VR study's LMER for 'Time to Escalator' for this study, i.e., $\text{TimeToEscalator} \sim \text{AtriaType} + \text{Block} + \text{VisibilityCondition} + \text{Session} + (1|\text{Participant})$. See Table 1 in the main text. We conduct this analyses using simulations with R's simr library. We ran 1000 simulations and compared the complete model against a model without the atria-type fixed effect. The atria-type manipulation was successful in producing statistically significant differences between the distributions of 'Average Destination Visibility', see Table S7 and Figure S2a. The observed effect size of -0.114 seconds fell outside the regime of practical significance, with a power of 5.8% for the group effect with a simulated Cohen d of 0.027. Notably, other effects, i.e., 'Visibility Condition' and 'Block', fulfilled the a priori power analysis assumptions with effects that were both practically and statistically significant.

A.2 Model fitness comparing linear, exponential, and threshold models

To estimate the relative quality of different models relating the 'Average Destination Visibility' and different wayfinding behavioral measures, Akaike Information Criteria (AIC) and Bayesian Information Criteria (BIC) were evaluated, the results are presented in table S2.

	'Time to Escalator'			'Avg. Vertical Head Movement'			'Avg. Cosine Similarity'		
	Linear	Exponential	Threshold	Linear	Exponential	Threshold	Linear	Exponential	Threshold
AIC	3368.08	**3365.33**	3377.39	-5969.44	-5992.94	**-6025.35**	-3499.08	-3496.23	**-3500.29**
BIC	3377.51	**3374.77**	3386.82	-5960.00	-5983.51	**-6015.91**	-3489.64	-3486.79	**-3490.85**
a	-100	29.73	29.88	-0.4103	0.0435	0.0451	2.1410	0.8183	0.8086
b	29.55	-4.03	25.03	0.0414	-18.19	0.0206	0.8169	2.3939	0.9148

Table S1. Regression results for the first analysis in the desktop VR study evaluated for linear, exponential, and threshold models using Akaike Information Criteria (AIC) and Bayes Information Criteria (BIC). The lowest AIC and BIC are formatted in bold. a and b are the parameters of the model, linear $f(x) = a \cdot x + b$, exponential $f(x) = a \cdot e^{b \cdot x}$, threshold $f(x) = a$ if $x = 0$ and $f(x) = b$ if $x > 0$

	'Time to Escalator'			'Avg.Vertical Head Movement'			'Avg. Cosine Similarity'		
	Linear	Exponential	Threshold	Linear	Exponential	Threshold	Linear	Exponential	Threshold
ΔAIC	2.75	**best**	12.06	55.91	32.41	**best**	1.21	4.06	**best**
$2 \times log(B_{10})$	2.02	**best**	4.98	8.047	6.96	**best**	0.38	2.80	**best**

Table S2. AIC and BIC comparison parameters

Across two of the wayfinding behavioral measures (i.e., 'Average Vertical Head Movement' and 'Average Cosine Similarity'), our conjecture of a nonlinear relationship between wayfinding and 'Average Destination Visibility' is confirmed. For 'Average Vertical Head Movement' and 'Average Cosine Similarity', the 'best' model is the Threshold model, as can be seen in Table S1. For 'Time to Escalator', our analysis shows that the Exponential model is the 'best' model.

To be able to evaluate the differences between the three models (Exponential, Threshold, Linear), a model fitness analysis was conducted. Specifically, we follow[3] comparing the absolute differences in AIC and[4] to evaluate the BIC criteria, see Table S1. The result of this analysis is nuanced. Although there is evidence for the nonlinear relationship between 'Average Destination Visibility' with the nonlinear models performing better than the linear model in all cases (see Table S1), this evidence is strong only in the case of 'Average Vertical Head Movement' Δ_iAIC > 10,[3], see Table S2. For the rest of the cases, the alternative models would have been acceptable options for the collected data regime as their difference is low. It is important to note the practical implications of the models. In contrast to the threshold model, the exponential and linear models would not be able to extrapolate well beyond the collected data regime, reaching unreasonable and practically impossible values beyond the measurement boundaries (e.g., Cosine Similarity greater than 1 and 'Time to Escalator' and 'Average Vertical Head movements' below humanly possible values).

A.3 LMER analysis model meta results

Complementary to the LMER analysis reported in the Results section in the main manuscript, Meta results are provided below for each of the three models (Table S3, Table S4 and Table S5.

Model:	MixedLM	Dependent Variable:	TimeToEscalator
No. Observations:	828	Method:	REML
No. Participants:	69	Scale:	40.8373
No. of Observations per Participant	12	Log-Likelihood:	-2765.7743
		Converged:	Yes

Table S3. 'Time to Escalator', Linear Mixed Effects Model Regression (LEMR) Meta Results for TimeToEscalator $\sim$ AtriaType + Block + VisibilityCondition + Session + (1|Participant)

Model:	MixedLM	Dependent Variable:	AvgVerticalHeadMovement
No. Observations:	828	Method:	REML
No. Participants:	69	Scale:	0.0005
No. of Observations per Participant	12	Log-Likelihood:	1877.2165
		Converged:	Yes

Table S4. 'Avg. Vertical Head Movement', Linear Mixed Effects Model Regression (LEMR) Meta Results for VerticalHeadMovement $\sim$ AtriaType + Block + VisibilityCondition + Session + (1|Participant)

Model:	MixedLM	Dependent Variable:	AvgCosineSimilarity
No. Observations:	828	Method:	REML
No. Participants:	69	Scale:	0.0087
No. of Observations per Participant	12	Log-Likelihood:	700.3615
		Converged:	Yes

Table S5. 'Avg. Cosine Similarity', Linear Mixed Effects Model Regression (LEMR) Meta Results for AvgCosineSimilarity $\sim$ AtriaType + Block + VisibilityCondition + Session + (1|Participant)

A.4 Non-parametric analysis to evaluate the effect of 'Visibility Condition' and atria-type on wayfinding

A.4.1 'Visibility Condition'

To evaluate the robustness of the significance in the difference in wayfinding performance between non-visible and visible destinations, we conducted a matched-data non-parametric hypothesis test in addition to the LMER analysis from the main text. For this test, we segment the data into two matched 'Visibility Conditions' (i.e., Non-Visible and Visible) and performed a two-tailed Wilcoxon signed-rank test.

Every NV trial was matched with its equivalent V trial. By equivalent, we are referring to a trial performed by the same participant during the same block, and with the destination on the same side of the entrance (left versus right). This grouping was mainly chosen for rigor, but the inclusion of the 8 trials with reduced 'Average Destination Visibility' and their matching visible trials did not affect the analysis.

A two-sided Wilcoxon Signed-rank test was performed to test for an effect of matched 'Visibility Condition' on each of the three wayfinding measures. Descriptive statistics for the non-visible and visible conditions for each wayfinding measure and the results of the statistical tests can be found in Table S6. Visualizations of the data and whisker box plots can be found in Figure 3 in the main text. These results confirm our LMER analysis results. There was a large ($r > 0.5$) and significant ($p < 0.001$) effect of the Destination's 'Visibility Condition' on each of the three wayfinding measures.

	'Time to Escalator' (s)		'Average Vertical Head Movement'		'Average Cosine Similarity'	
	NV	V	NV	V	NV	V
Median	27.3353	23.3515	0.0361	0.0128	0.8398	0.9538
Mean	29.8842	24.6013	0.0451	0.0198	0.8086	0.9206
Std	8.9815	3.5348	0.0305	0.027	0.1407	0.0838
W	7468		7136		6884	
r	0.502		0.507		0.511	
Z	-14.304		-14.444		-14.551	
p	**<0.001** ***		**<0.001** ***		**<0.001** ***	

Table S6. Descriptive statistics and the results of the two-tailed matched Wilcoxon signed-rank test for non-visible (NV) and visible (V) groups. *=Statistically significant after Bonferroni correction

A.4.2 Atria-type

	ADV		TE (s)		VHM		ACS	
	Distributed	Centralized	Distributed	Centralized	Distributed	Centralized	Distributed	Centralized
Median	0.026645	0.058738	23.315185	23.408570	0.011063	0.016550	0.956200	0.951897
Mean	0.027843	0.057993	24.763498	24.578964	0.018347	0.021605	0.919228	0.919335
Std	0.009925	0.007910	4.534209	2.848100	0.018953	0.022207	0.09552	0.07721
U	567.0		20568.0		19047.0		20452.0	
r	0.844		0.057		0.026		0.061	
Z	-17.172		-1.170		-2.232		-1.24	
p	**<0.001** ***		0.242		0.110		0.213	

Table S7. Descriptive statistics and the results of the two-tailed Mann-Whitney U test for the distributed and the centralized atria types. With the exception of 'Average Destination Visibility', all of these results are null (i.e. $p > 0.001$).'Average Destination Visibility' (ADV), 'Time to Escalator' (TE), 'Average Vertical Head Movement' (AVHM), 'Average Cosine Similarity' (ACS).

With respect to environmental geometry, we observed a large (r>0.5) statistically significant difference (p<0.001) in 'Visibility Condition' for the 'entrance-forward-facing' destinations on the second floor between the distributed and centralized atria types (see Figure S2a). Please note that 'Visibility Condition' (i.e., non-visible and visible) is derived from the 'Average Destination Visibility'. The descriptive statistics for 'Average Destination Visibility' (restricted to destinations 6 and 7) are for the distributed atria-type, mean=0.028, median=0.027, std=0.0099, and for the centralized atria, mean=0.058, median=0.059, std=0.008. A two-tailed Mann-Whitney U test shows that there is a significant effect (U = 567, Z = -17.17, p < 0.001, r = 0.84).

	Distributed		Centralized	
Destination	6	7	6	7
Median	0.034600	0.022343	0.061057	0.057275
Mean	0.034771	0.020916	0.059496	0.056491
Std	0.008622	0.005172	0.008996	0.006346

Table S8. 'Average Destination Visibility' statistics for destinations 6 and 7 ('entrance-front-facing', second floor), see figure S2a

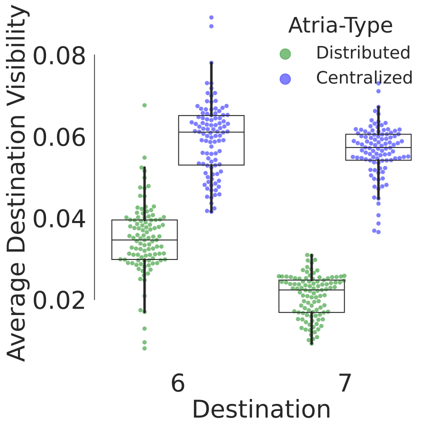

(a) Distribution of 'Average Destination Visibility' for 'entrance-forward-facing' destinations on the second floor.

Figure S2. Group effects. Software used to create this figure: Python[5] (version 3.5.8), https://www.python.org/downloads/release/python-358/; Seaborn[6] (version 0.11.1), https://seaborn.pydata.org/index.html; Matplotlib[7] (version 3.3.2), https://matplotlib.org/3.3.2/users/installing.html

A.5 Inferential statistics for wayfinding performance towards first-floor destinations

Our main analysis included the analysis of wayfinding towards second-floor destinations. For completeness, we provide descriptive statistics for wayfinding performance towards first-floor destinations. Table S9 provides descriptive statistics for time taken to reach the destination (not the bottom of the escalator). Table S10 provides descriptive statistics for 'Average Destination Visibility' towards first floor destinations.

	mean	std	min	max
target (destination)				
1	17.710089	13.675788	8.069824	134.00240
2	42.360579	2.373105	39.541990	56.36572
3	43.038635	2.457392	39.994870	57.09180
4	18.016306	13.379453	8.683838	102.67070

Table S9. Descriptive statistics for total time taken to find first floor destination across groups

target (destination)	mean	std	min	max
1	9.448496	3.368947	2.026758	31.296824
2	4.092731	0.464197	1.272471	5.739927
3	4.229682	0.502384	1.372993	7.993843
4	9.709349	3.113799	2.235815	17.215324

Table S10. Descriptive statistics for ‘Average Destination Visibility’ towards first floor destination across groups

A.6 Exemplary screenshots from the desktop VR study

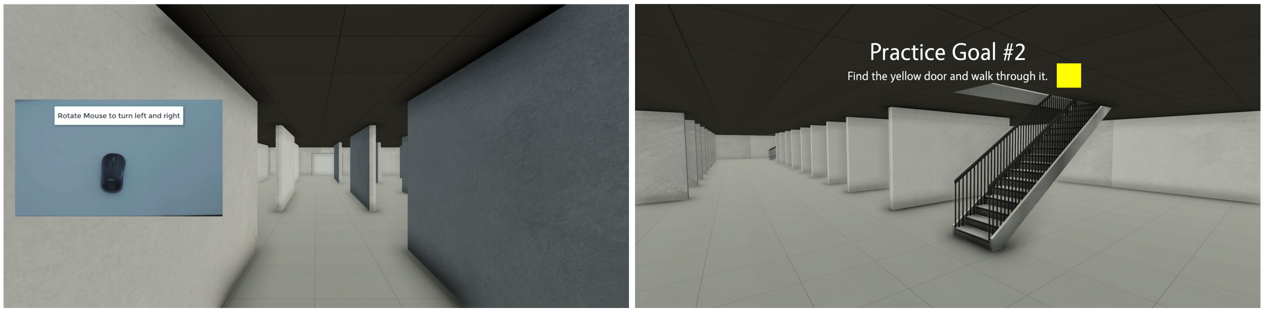

(a) An interactive tutorial to review possible movements and rotations using the mouse and keyboard

(b) One of four practice trials where participants were instructed to find a doorway with a specific color

Figure S3. Exemplary screenshots from the desktop VR study (captured from the Unity3D[8] game engine) showing the environment used for the training scene that was different from the actual test environments. During training, participants reviewed possible movement options and were introduced to the task structure (i.e., find a door with a specific color). Software used to create these figures: Unity3D[8] (version 2018.4.16f1), https://unity3d.com/unity/whats-new/2018.4.16f1

B Study 2: Visibility-Based Cognitive Agents

B.1 Agent analysis LMER model meta results

Complementary to the LMER analysis reported in the Results section in the main manuscript, Meta results are provided below for each of the three models.

Model:	MixedLM	Dependent Variable:	diffTime
No. Observations:	3200	Method:	REML
No. Groups:	16	Scale:	1.6451
Min. group size:	200	Log-Likelihood:	-5388.2481
Max. group size:	200	Converged:	Yes
Mean group size:	200.0		

Table S11. ‘Difference in ‘Time to Escalator’, Linear Mixed Effects Model Regression (LEMR) Results for $\text{diffTime} \sim \text{AgentType} + (1|\text{Agent})$

Model:	MixedLM	Dependent Variable:	diffCos
No. Observations:	3200	Method:	REML
No. Groups:	16	Scale:	0.0053
Min. group size:	200	Log-Likelihood:	3803.1725
Max. group size:	200	Converged:	Yes
Mean group size:	200.0		

Table S12. ‘Difference in Average Cosine Similarity’, Linear Mixed Effects Model Regression (LEMR) Results for diffCos $\sim$ AgentType + (1|Agent)

B.2 Non-parametric analysis of the differences between agents and humans

	Diff. ‘Time to Escalator’		Diff. ‘Average Cosine Similarity’		DTW Trajectory Distance	
	Cognitive	Shortest Path	Cognitive	Shortest Path	Cognitive	Shortest Path
Median	2.7597	4.8066	0.0451	0.0754	0.6818	1.1856
Mean	2.5995	4.9189	0.0459	0.1516	0.9736	1.661
Std	1.1724	3.1868	0.0348	0.1501	0.9750	0.3474
W	55243.5		50826.0		248547.0	
r	0.5249		0.5385		0.3484	
Z	-29.6956		-30.4597		-19.7099	
p	**<0.001** ***		**<0.001** ***		**<0.001** ***	

Table S13. Descriptive statistics and the results of two-tailed matched Wilcoxon signed-rank tests for the differences between agents (visibility-based cognitive vs shortest-path agents) and humans.

B.3 Dynamic Time Warping

In addition, we used Dynamic Time Warping (DTW) to quantify differences in the ‘Spatial Distribution of Paths’. DTW allows comparisons of two time-series sequences with varying lengths and speeds to derive a distance metric between two sequences (e.g., movement trajectories) and to calculate an average path. ‘DTW distance’ between human trajectories and trajectories of the visibility-based cognitive agents was reduced when compared to the corresponding trajectories of shortest-path agents (i.e., without visibility). Next, we calculated the average DTW trajectories of the humans and compared the average DTW distance of all the individual agent trajectories to humans’ average trajectory per trial (see Figure S4a).

We statistically evaluated whether the fitness of ‘Time to Escalator’, ‘Average Cosine Similarity’, and DTW distances for the visibility-based cognitive agent model was better than the fitness of these measures for the shortest-path agent model using matched two-tailed Wilcoxon signed-rank tests.

The results revealed that the improvements in ‘Time to Escalator’ and ‘Average Cosine Similarity’ observed in cognitive agents were statistically significant ($p < 0.001$). Large effects were seen for ‘Time to Escalator’ and ‘Average Cosine Similarity’ (r>0.5), but the comparison of ‘DTW Distances’ showed only a moderate effect size (r>0.3). Descriptive and inferential statistics for these comparisons can be found in Table S13

In terms of ‘DTW distance’, we found that the visibility-based cognitive agents were able to replicate patterns of human behavior in the desktop VR experiment for the measures ’Time to Escalator’ and ’Average Cosine Similarity’. In contrast, shortest-path agents, who were agnostic to the visibility of destinations, were unable to replicate this pattern.

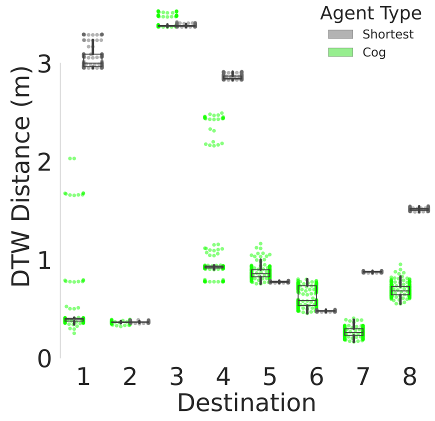

(a) Average Dynamic Time Warped (DTW) Distance between the agents' trajectories and the average human trajectory.

Figure S4. Differences between agents and humans in terms of three wayfinding behavioral measures. Software used to create this figure: Python[5] (version 3.5.8), https://www.python.org/downloads/release/python-358/; Seaborn[6] (version 0.11.1), https://seaborn.pydata.org/index.html; Matplotlib[7] (version 3.3.2), https://matplotlib.org/3.3.2/users/installing.html

B.4 A state-machine model of the visibility-based cognitive agent

The main text of this article presents an overview of the design behind the visibility-based cognitive agent. Figure S5 complements this description with a schematic view of the agent as a state machine.

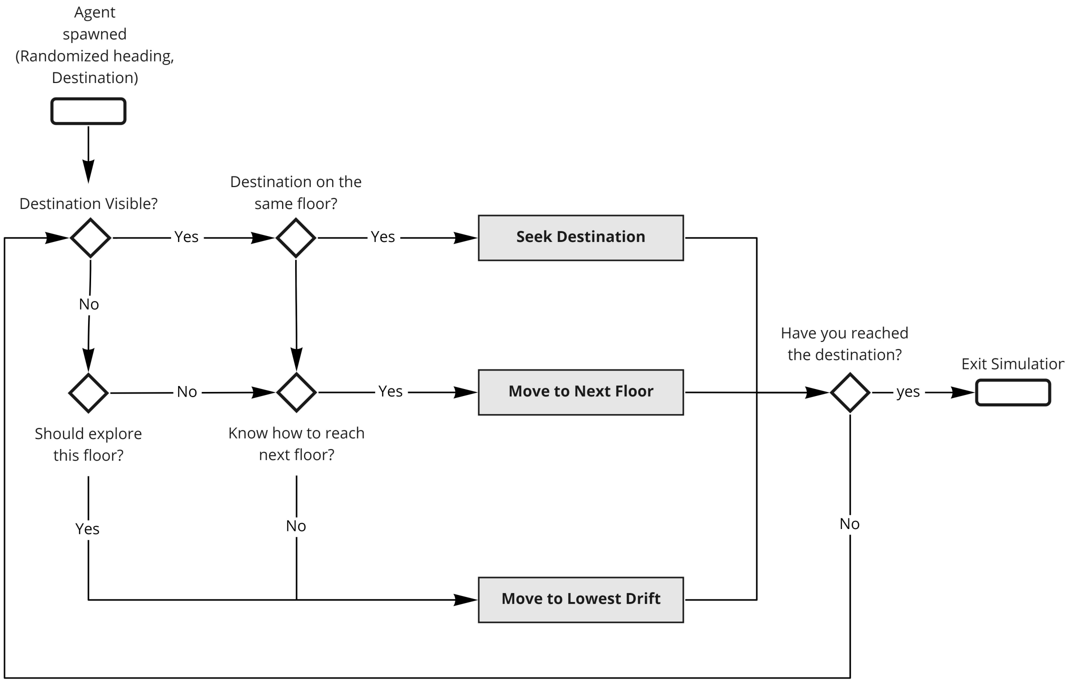

Figure S5. A state machine diagram depicting agents' behavioral model. Software used to create this figure: Miro (2021)[9], https://miro.com/index/

FSM is an abstract machine that can be in exactly one of a finite number of states at any given time. The FSM can change from one state to another in response to some input. The change from one state to another is called a transition. An FSM is defined by a list of its states, its initial state, and the inputs that trigger each transition.

The diagram 'Should explore this floor?' relates to the 'sufficiently explored' term in the agent description. The implementation of this behavior is achieved by segmenting the space in 'Zones', which are discrete volumetric segments of space.

An agent is able to perceive these zones and keeps track of which zones it has seen and which zones it has visited (physically positioning itself within that zone). If an agent finds itself in a visited zone more than tr_v times (currently set to 1) it turns around to try to perceive other zones and destinations. The agent has an 'anxiety counter' which is increased when revisiting a zone. If the anxiety counter is above a certain threshold tr_a (currently set to 2) and no new zones are available on the same floor, the decision to move to the next floor is taken. This algorithm is based solely on the author's qualitative observation of the VR participants and intended as a working definition of 'sufficiently explored'. A more adequate implementation of this algorithm and its validation is outside the scope of this paper.

B.5 The simulation environment

Grid cell locations were pre-computed in the virtual environment on the basis of environmental geometry, distinguishing walkable versus non-walkable cells, that was used to generate a lattice to supports agents' routing (cell dimensions of $0.25m \times 0.25m$) and drift perception (cell dimensions of $0.5m \times 0.5m$).

A ray-casting method was used to perform this calculation, taking a horizontal view angle of 150°, a vertical view angle of 100°, and a view range of 100 meters. These view settings mimicked humans' view angles within the virtual environment setup (i.e., camera settings of the FPC used in the desktop VR study considering observed head movements).

C Data analysis

C.1 The dataset

The results of the Desktop VR study (i.e., study 1) and the simulation experiments (i.e., study 2) are provided in the accompanying dataset (see Data availability section), see figure S6. Each CSV file represents a single trial, see table S14. The filename includes the metadata of the specific trial, X is the participant or agent identifier, Y is the group (atria-type, 1. Distributed, 2. Centralized) identifier, Z trial number, and W the target number (destination).

```
Data
├── Agents
│   ├── ShortestPathAgents
│   │   └── SimulationData
│   │       ├── Sim_X_group_Y_trial_Z_target_W.csv
│   │       └── ...
│   └── VisibilityBasedCognitiveAgents
│       └── SimulationData
│           ├── Sim_X_group_Y_trial_Z_target_W.csv
│           └── ...
└── Humans
    ├── Session1
    │   ├── Data_X_group_Y_trial_Z_target_W.csv
    │   └── ...
    └── Session2
        ├── Data_X_group_Y_trial_Z_target_W.csv
        └── ...
```

Figure S6. Folder structure of the accompanying dataset. Software used to create this figure: Latex (Version TexLiveVersion 2020, Overaleaf), https://www.overleaf.com/blog/tex-live-2020-now-available
.

Each CSV file (i.e. trial) contains n points measured over the trajectory of a participant. For every point we measure the 'Time' since the start of the trial, the 'Position' in the VR environment, and the camera angles corresponding to participants' horizontal (yaw) and vertical (pitch) camera rotations, i.e., 'View azimuth' and 'View elevation'. Additionally, the last column represents the 'Visibility' denoting how much of the target (destination) was in their field of view, which is further explained in section C.4 below.

	Time	Position	View azimuth	View elevation	Visibility
1	t_0	$(x_0; y_0; z_0)$	a_0	e_0	v_0
			...		
i	t_i	$(x_i; y_i; z_i)$	a_i	e_i	v_i
			...		
n	t_n	$(x_n; y_n; z_n)$	a_n	e_n	v_n

Table S14. Data schema for each trial (i.e., a single CSV file)

.

C.2 Time to Escalator

'Time to Escalator' was calculated as the time participants took to reach the escalator. The reported y_i value corresponds to the position of participants' camera height $1.7m$ at time i. To identify the point where the escalator was reached we evaluate the moment when participants' camera's height exceeded $y > 2m$, i.e. the first step of the stairs, see figure S7. The time entry t_i at that point is considered the 'Time to Escalator' for that trial. For the rest of the behavioral measures explained below, we truncate the data until this point, referred to as the analysis window.

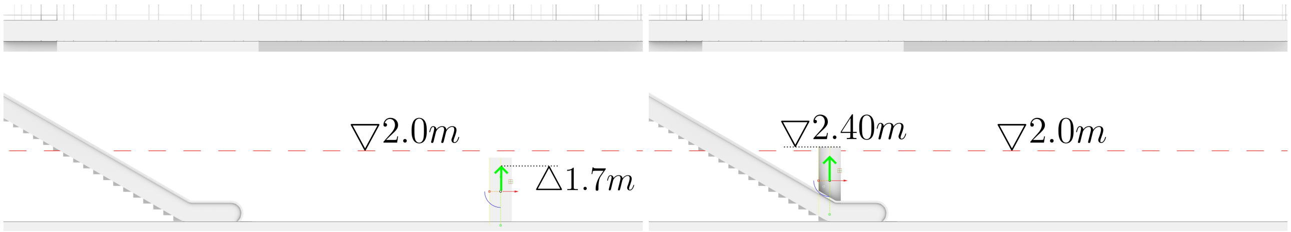

(a) t_j **(b)** Time To Escalator $= t_i$

Figure S7. The illustration of two time steps, time step j showing an agent or a participant on the ground floor before reaching the escalator and time step i showing an agent or a participant reaching the escalator once their camera height exceeds $y > 2m$. Software used to create these figures: Rhino 6 for Windows (Version 6)[10], https://www.rhino3d.com/download/

.

C.3 Vertical Head Movement

Here we calculate 'Vertical Head Movement' by measuring the angular difference in participants' view perspective up/down rotations (i.e., pitch) at consecutive path points, $n-1$ angular differences. $h_i = \alpha_{i+1} - \alpha_i$, and $\text{AverageVerticalHeadMovement} = \sum^{n-1} h_i/(n-1)$

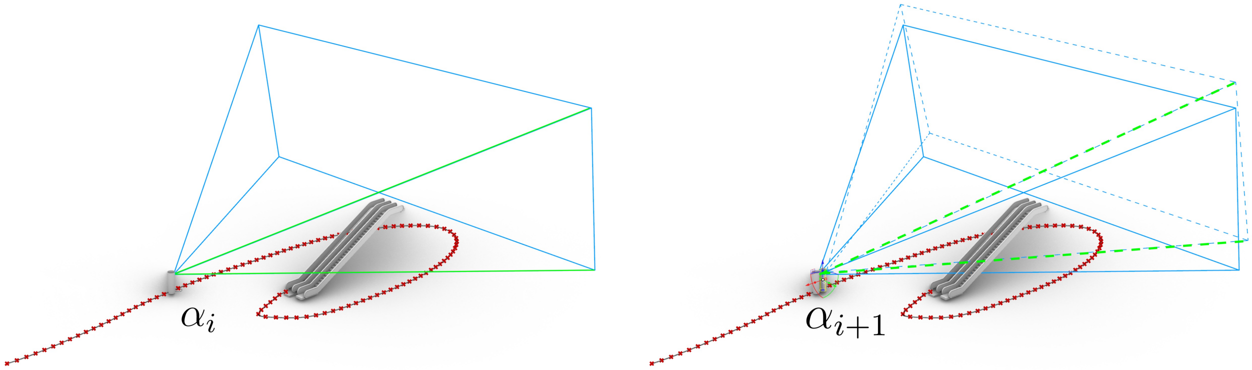

(a) The vertical angle, α_i, of a participant's field of view at P_i **(b)** The vertical rotation angle, α_{i+1}, of a participant's field of view at P_{i+1}

Figure S8. Vertical Head Movement is calculated as the difference between consecutive vertical camera rotation angles. Software used to create these figures: Rhino 6 for Windows (Version 6)[10], https://www.rhino3d.com/download/

C.4 Destination Visibility

The ‘Destination Visibility’ column of the dataset is calculated by casting m rays from each position along participants’ path within their 3D field of view towards the destination (i.e. doorway). We denote ‘Destination Visibility’, v_i, as the percentage of rays that hit the target (destination). Specifically, we distinguish rays that hit the target as r_i^h and rays that miss the target as r_i^m. The ‘Destination Visibility’ at a point P_i is calculated as $v_i = (r^h/(r^m + r^h)) \cdot 100$. The ‘Average Destination Visibility’ reported is $\text{AverageDestinationVisibility} = \sum^n v_i/(n)$

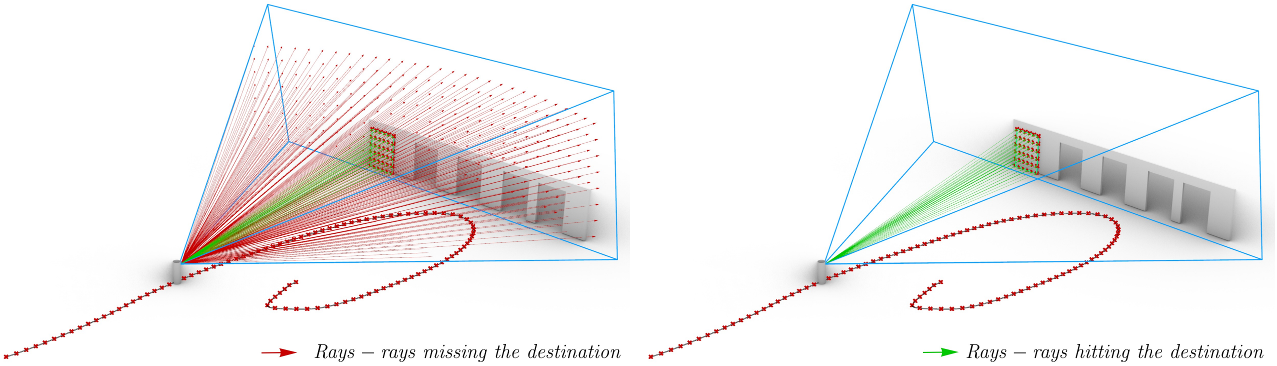

Figure S9. An illustration of the visibility percentage calculation. Figure (a) shows the total amount of rays (red and green) cast from participant’s-height within their field of view. Rays that miss the target are colored in red. Figure (b) shows only the rays that hit the target (i.e., destination doorway) in green. Software used to create these figures: Rhino 6 for Windows (Version 6)[10], https://www.rhino3d.com/download/

C.5 Cosine Similarity

To calculate the ‘Cosine Similarity’ we start by calculating the unitary direction of participants’ movement vector $VP_i = P_{i+1} - P_i$ and $\hat{VP_i} = VP_i/\|VP_i\|$. We then calculate the vector towards the escalator $VE_i = PE - VP_i$ and its unitary vector $\hat{VE_i} = VE_i/\|VE_i\|$. The cosine similarity c is the dot product between these two vectors $c = \hat{VP_i} \cdot \hat{VE_i}$. We report ‘Average Cosine Similarity’ being the average over the $n-1$ vectors, $\text{AverageCosineSimilarity} = \sum^{n-1} c_i/(n-1)$. For an illustration of this process, see figure S10. ‘Average Cosine Similarity’ ranges from ‘-1’ to ‘1’, where ‘1’ means that participants were heading directly towards the escalator, and ‘-1’ means that they were heading in the opposite direction.

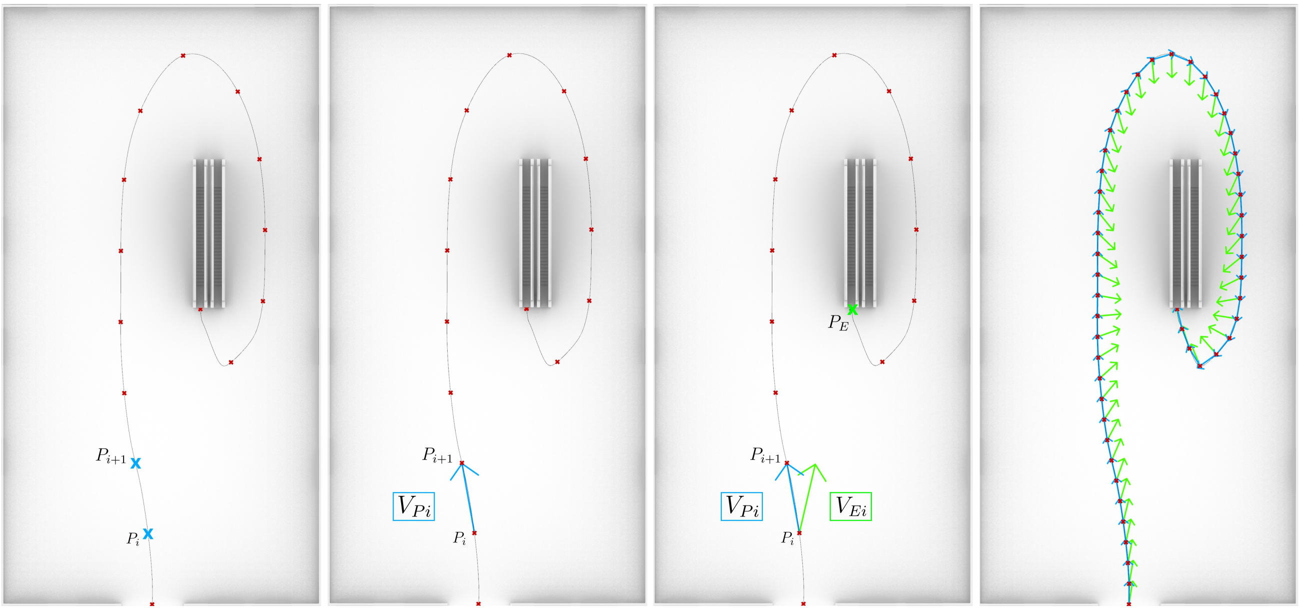

(a) Two consecutive points along a participant's path $[P_{i+1}, P_i]$ used to calculate the direction of the participants movement vector, VP_i.

(b) The direction of a participant's movement vector VP_i at point P_i is shown in blue.

(c) The two unscaled vectors $[VP_i, VE_i]$ used for the calculation of the cosine similarity at point P_i. The point reached at the escalator PE is shown with a green X mark.

(d) A visualization of multiple movement vectors (blue) along a participant's path and the corresponding vector towards the bottom of the escalator(green).

Figure S10. A visualization of vectors used to calculate cosine similarity along participants' path for the analysis window (please note that these illustrations are exemplary and thus do not represent vectors at the correct scale. In addition, the spacing between the measured points along a path is not representative. Software used to create these figures: Rhino 6 for Windows (Version 6)[10], https://www.rhino3d.com/download/

References

1. R Core Team. *R: A Language and Environment for Statistical Computing, (version 3.6.3 2020-02-29).* R Foundation for Statistical Computing, Vienna, Austria (2020).
2. Green, P. & MacLeod, C. J. simr: an r package for power analysis of generalised linear mixed models by simulation. *Methods Ecol. Evol.* **7**, 493–498, DOI: 10.1111/2041-210X.12504 (2016).
3. Burnham, K. P. & Anderson, D. R. Multimodel inference: understanding aic and bic in model selection. *Sociol. methods & research* **33**, 261–304 (2004).
4. Kass, R. E. & Raftery, A. E. Bayes factors. *J. american statistical association* **90**, 773–795 (1995).
5. Van Rossum, G. & Drake Jr, F. L. *Python reference manual, (version 3.5.8)* (Centrum voor Wiskunde en Informatica Amsterdam, 1995).
6. Waskom, M. L. seaborn: statistical data visualization,(version 0.11.1). *J. Open Source Softw.* **6**, 3021, DOI: 10.21105/joss.03021 (2021).
7. Hunter, J. D. Matplotlib: A 2d graphics environment, (version 3.3.2). *Comput. Sci. & Eng.* **9**, 90–95, DOI: 10.1109/MCSE.2007.55 (2007).
8. Unity, (version 2018.4.16f1). *Unity Game Engine*.
9. Miro. *Miro*.
10. McNeel, R. *et al.* Rhinoceros 3d, version 6.0. *Robert McNeel & Assoc. Seattle, WA* (2021).
